# Self‐Assembling Hybrid Hydrogel Reprograms the Stromal Vascular Fraction to Treat Osteoarthritis

**DOI:** 10.1002/advs.202517297

**Published:** 2026-04-20

**Authors:** Waifang Hou, Yongfeng Chen, Longyou Xiao, Wei Qi, Tianshu Du, Qiang Sun, Borui Xue, Qinghe Zhao, Pengfei Xie, Zi Ye, Fei Han, Lingli Guo, Yang Jiao, Ranran Zhang, Hong Jia, Xiao Huang, Jianwei Guo, Peng Wang, Huayi Wang, Yuanrui Wang, Wenjing Chen, Haining Wu, Liumin He, Dawei Zhang

**Affiliations:** ^1^ Department of Orthopaedics Xijing Hospital The Fourth Military Medical University Xi'an P. R. China; ^2^ Department of Spine Surgery The Third Affiliated Hospital Sun Yat‐Sen University Guangzhou P. R. China; ^3^ Air Force 986(th) Hospital The Fourth Military Medical University Xi'an P. R. China; ^4^ Department of Nursing Air Force Medical University Xi'an P. R. China; ^5^ Re‐Stem Biotechnology Co. Ltd Suzhou P. R. China; ^6^ Lintong Rehabilitation and Convalescent Centre of the Joint Logistics Support Force Xi'an P. R. China

**Keywords:** hydrogel, osteoarthritis, stemness, stromal vascular fraction

## Abstract

Osteoarthritis (OA) is characterized by progressive cartilage degradation accompanied by limited intrinsic repair capacity. Stromal vascular fraction (SVF) transplantation has demonstrated potential for regenerating damaged joint tissue; however, the pathological microenvironment impairs SVF stemness, thereby limiting therapeutic efficacy. Herein, we developed a bioinspired hydrogel formed via the cross‐linking of hyaluronic acid‐grafted dopamine and a fibroblast growth factor 2‐mimetic peptide‐modified RADA16‐I. This injectable hydrogel combined excellent gelation performance with multifunctionality, particularly in enhancing SVF proliferation and chondrogenic differentiation. The hydrogel activated forkhead box M1 (FOXM1) – mediated epigenetic reprogramming, enhancing DNA repair capacity and increasing chromatin accessibility at pluripotency loci. In a rat OA model, combined hydrogel and SVF transplantation significantly enhanced articular cartilage regeneration and ameliorated OA symptoms. This study provides preliminary evidence showing that a biomaterial‐mediated epigenetic reprogramming strategy improves recovery in OA, highlighting the therapeutic potential of the novel hydrogel for stem cell‐based regenerative medicine.

## Graphical abstract

This study presents a bioinspired injectable hydrogel that enhances the therapeutic potential of stem cell‐rich stromal vascular fraction for treating osteoarthritis. By reprogramming cell behavior through epigenetic modulation, the hydrogel promotes cartilage regeneration and reduces joint damage in a rat model, offering a promising new approach for stem cell‐based tissue repair.

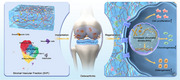

## 1 Introduction

Osteoarthritis (OA), a degenerative joint disease, is pathologically characterized by cartilage degradation, subchondral bone remodeling, and synovial inflammation [1]. Achieving functional cartilage regeneration is a pivotal therapeutic objective in OA management. However, the intrinsic regenerative capacity of cartilage is fundamentally constrained by its avascular nature, low cellularity, and the absence of endogenous stem cell recruitment mechanisms [2]. This has shifted the focus to mesenchymal stem cell (MSC)‐based therapies, which synergistically integrate stem cell differentiation capacity, immunomodulatory properties, and the regenerative potential of the extracellular matrix (ECM) to promote cartilage repair [3, 4].

The stromal vascular fraction (SVF), which is rich in clinically relevant adipose‐derived mesenchymal stem cells (ADSCs), demonstrates promising therapeutic potential for OA, bone defects, and soft tissue regeneration [5, 6, 7, 8]. The therapeutic benefits of SVF are primarily derived from its minimal immunogenicity, robust regenerative capacity, and anti‐inflammatory properties [9]. Despite preclinical studies demonstrating the efficacy of SVF therapies in primates, particularly humans [10], recent studies indicate diminished stem cell populations and regenerative potential when exposed to the hostile intra‐articular inflammatory microenvironment [11, 12].

Advances in bioactive scaffolds have shown great potential for use in stem cell reinforcement therapy [13, 14, 15, 16]. An emerging strategy is to use cell‐signaling materials to mimic the fibrillar components of natural ECMs. Functional nanostructures and viscoelastic materials can mediate multiple aspects of stem cell behavior [17, 18]. RADA16‐I [Ac‐(RADA)_4_‐CONH_2_], a self‐assembling peptide (SAP), consists of four repeats of arginine (R), alanine (A), aspartic acid (D), and alanine (A) [19]. Its alternating ionic hydrophilic and hydrophobic structure enables the spontaneous formation of a nanofiber‐based 3D macroscopic hydrogel in physiological media or saline, without the need for exogenous cross‐linkers, avoiding the use of toxic/non‐degradable chemicals. Owing to its excellent biocompatibility, inherent biodegradability, and facile functionalization capability, RADA16‐I is an ideal base material for constructing multifunctional hydrogels tailored for tissue engineering applications. The N‐terminus of RADA16‐I can be chemically or genetically conjugated to aromatic peptides, whereas its C‐terminus accommodates bioactive motifs, such as RGD or IKVAV [20, 21]. Functionalization of the N‐and C‐termini of RADA16‐I with specific bioactive peptide sequences preserves its self‐assembly while conferring native peptide functions (e.g., RGD‐mediated adhesion or IKVAV‐induced neurite guidance) [22, 23]. These advantages further enable the combination with other biomimetic hydrogels through blending or interpenetrating polymer network formation, thereby enhancing both the mechanical and bioactive properties. Particularly, the structure of aromatic‐rich hyaluronic acid‐grafted‐dopamine (HADA) facilitates reversible non‐covalent interactions (π‐π stacking, cation‐π, catechol hydrogen bonds) with RADA16‐I F‐SAP [24, 25]. Furthermore, HADA hydrogel's covalent crosslinks arise from catechol oxidation to quinones, eliminating the need for xenobiotic crosslinkers [26]. The dynamic bonds between quinone and catechol provide self‐healing properties, while the versatility of catechol permits tailored hydrogel designs. Thus, multifunctional HADA hydrogels provide structural support for combined F‐SAP applications, establishing them as highly translatable biomedical implants.

**SCHEME 1:**
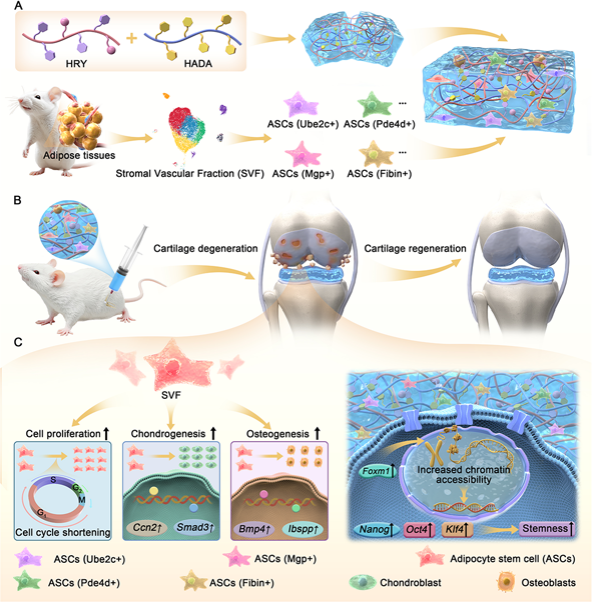
Schematic illustration of the HADA@HRY hydrogel and its therapeutic function in osteoarthritis treatment by enhancing stromal vascular fraction (SVF) stemness. (A) HADA@HRY hydrogel formulation and SVF incorporation. (B) Implantation of HADA@HRY hydrogel‐encapsulated SVF cells to promote joint repair in osteoarthritis. (C) HADA@HRY hydrogel enhances SVF stemness through forkhead box M1‐mediated epigenetic reprogramming, synergistically promoting chondrogenesis and osteogenesis for joint repair.

Herein, we present a bioinspired HADA@HRY hybrid hydrogel with a dual‐cross‐linking mechanism, fabricated through the covalent conjugation of HADA with the designer peptide HRY. The diverse components of this hydrogel emulate the ECM microenvironment and provide a biocompatible and cell‐promoting platform for SVF application in OA [27]. The findings of this study provide a promising therapeutic paradigm for OA treatment (Scheme 1).

## 2 Results

### 2.1 Design, Synthesis, and Characterization of the HADA@HRY Composite Hydrogel

**FIGURE 1:**
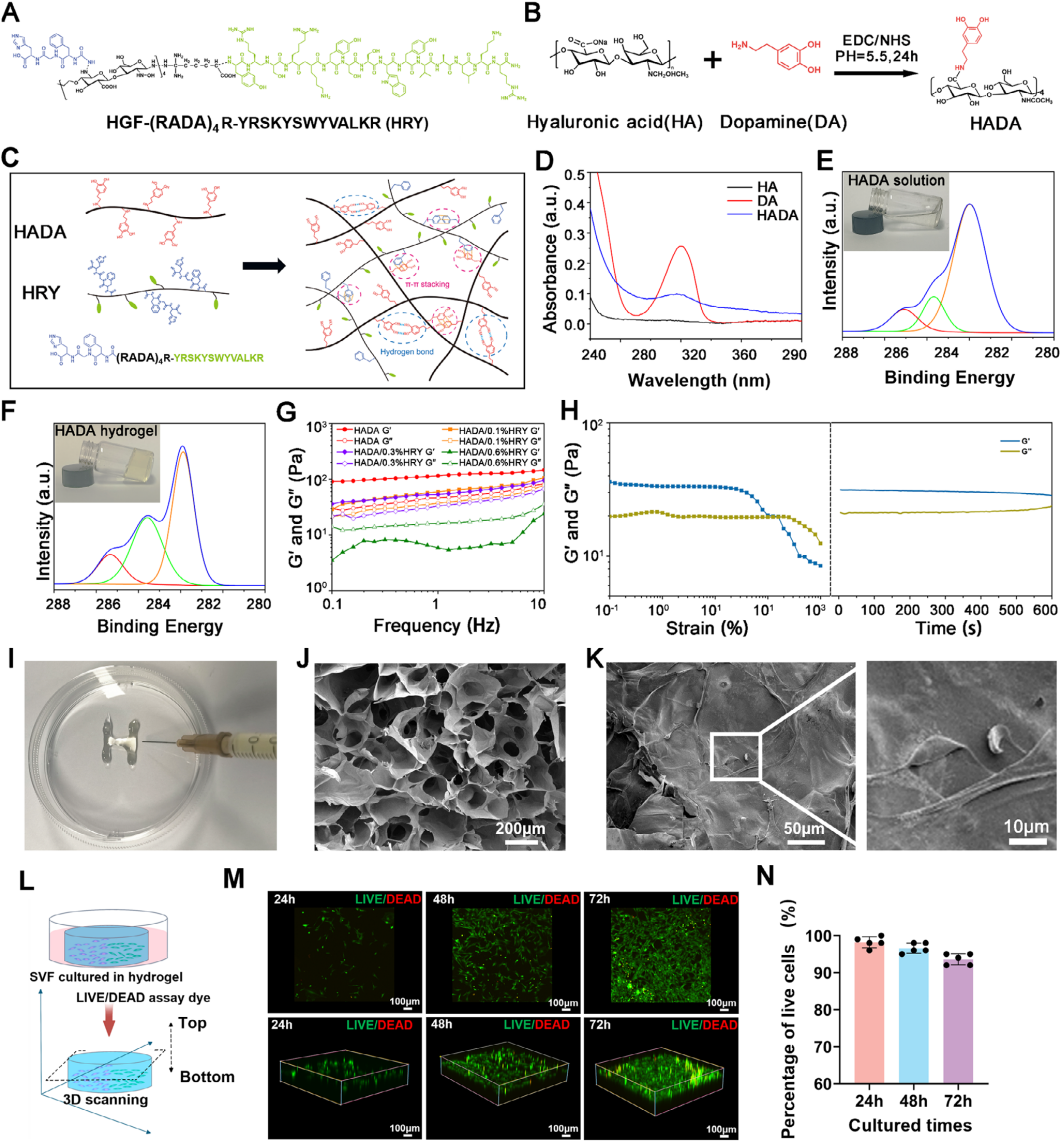
Characterization and biocompatibility of HADA@HRY. A) Structure of the designed self‐assembling peptide. B) Synthesis of HADA via EDC/NHS‐mediated grafting of DA to HA. C) Proposed intramolecular and intermolecular interactions between HADA and HRY. D) UV–vis spectra confirmed the successful synthesis of HADA. E‐F) Photographs of HADA solution/hydrogel and XPS C1s spectra. G) Rheology of HADA and HADA@HRY hydrogels (0.1‐10 s^−1^). H) Moduli (G′ & G′′) of HADA@HRY hydrogel vs. frequency at 1% γ: strain amplification (left) and recovery post‐1,000% strain (right). I) Injectability of HADA@HRY hydrogel: Smooth extrusion through a 1 mL syringe. J) SEM image of HADA@HRY hydrogel. K) HADA@HRY hydrogel morphology with SVF cell adhesion. L) Biocompatibility visualization: 3D confocal image of SVF‐hydrogel. M) Live/dead staining of SVF in HADA@HRY hydrogel (24/48/72 h). N) Quantified survival of encapsulated SVF (24/48/72 h; n = 5).

To develop a biocompatible and injectable hydrogel suitable for the complex microenvironment of the joint cavity, we synthesized a composite hydrogel by combining HADA with a self‐assembling peptide, HRY (Figure 1A). HADA was prepared via EDC/NHS‐mediated conjugation of dopamine (DA) with hyaluronic acid (HA) (Figure 1B). Successful grafting was confirmed using UV–vis spectroscopy, which showed a characteristic absorption peak at around 280 nm, corresponding to the aromatic ring of DA (Figure 1D). X‐ray photoelectron spectroscopy (XPS) further confirmed an enhanced signal at 285.5 eV, consistent with C═O (quinone) formation following gelation (Figure 1E, F), indicating oxidative conversion of catechol groups.

Although HA and its derivatives are inherently biocompatible, their limited biological activity and insufficient capacity to support cellular and tissue regeneration hinder their potential applications in tissue engineering. To enhance the potential of the hydrogel for stemness promotion, we incorporated a stemness‐inducing motif YRSKYSWYVALKR into the HRY peptide sequence. Mechanical characterization of the HADA@HRY composite hydrogels revealed a concentration‐dependent response to peptide incorporation. As the HRY concentration increased (0%, 0.1%, 0.3%, 0.6% w/v), distinct changes in rheological properties were observed (Figure 1G). Both HADA@0.1%HRY and HADA@0.3%HRY hydrogels exhibited gel‐like behavior, with a storage modulus (G′) greater than the loss modulus (G′′). Notably, HADA@0.3%HRY demonstrated enhanced mechanical stability relative to HADA@0.1%HRY. However, at a higher peptide concentration of 0.6%, the hydrogel transitioned to a sol‐like state (G′′ > G′), indicating disruption of the network structure and loss of mechanical integrity. These observations suggest that intermolecular interactions between HADA and HRY stabilize the cross‐linked architecture within a specific concentration range. Meanwhile, the HADA@0.3%HRY hydrogel displayed pronounced shear‐thinning behavior and rapid storage modulus recovery under cyclic strain, mimicking the viscoelastic properties required to withstand mechanical stress in inflamed joints (Figure 1H).

This behavior arises from a dual‐cross‐linking mechanism, wherein covalent cross‐linking is achieved through the oxidation of catechol groups in HADA, whereas dynamic physical cross‐linking is mediated by π‐π stacking interactions between phenyl groups in HADA and aromatic residues in HRY. Additional intramolecular and intermolecular hydrogen bonding further contributes to the physical cross‐linking network. These reversible, non‐covalent interactions confer robust self‐healing properties to the hydrogel (Figure 1C). Moreover, the spontaneous polymerization of DA derivatives reinforces the mechanical strength of the hydrogel. Thus, a hydrogel with enhanced mechanical properties was successfully synthesized through a one‐pot synthesis method. The HADA@HRY hydrogel exhibited excellent injectability, allowing for facile extrusion through a 1 mL syringe without clogging or fragmentation (Figure 1I), a critical property for minimally invasive intra‐articular administration. Scanning electron microscopy (SEM) revealed a uniform porous microarchitecture that was favorable for nutrient exchange and cellular infiltration (Figure 1J). The hydrogel supported adhesion and survival of SVF cells in vitro, with cells visibly attached and spread on the hydrogel surface (Figure 1K), suggesting favorable cytocompatibility. A biocompatibility assessment of the HADA@HRY hydrogel as a 3D carrier for SVF cell delivery was performed through comprehensive viability assays (Figure 1L). Confocal microscopy demonstrated excellent cell viability and stable proliferation within HADA@HRY hydrogels after 24 h (98.2% ± 1.48%), 48 h (96.6% ± 1.34%), and 72 h (93.6% ± 1.52%) of 3D culture conditions, indicating minimal cytotoxicity and confirming outstanding biocompatibility (Figure 1M,N).

### 2.2 HADA@HRY Promotes the Proliferation of SVF Cells In Vitro

**FIGURE 2:**
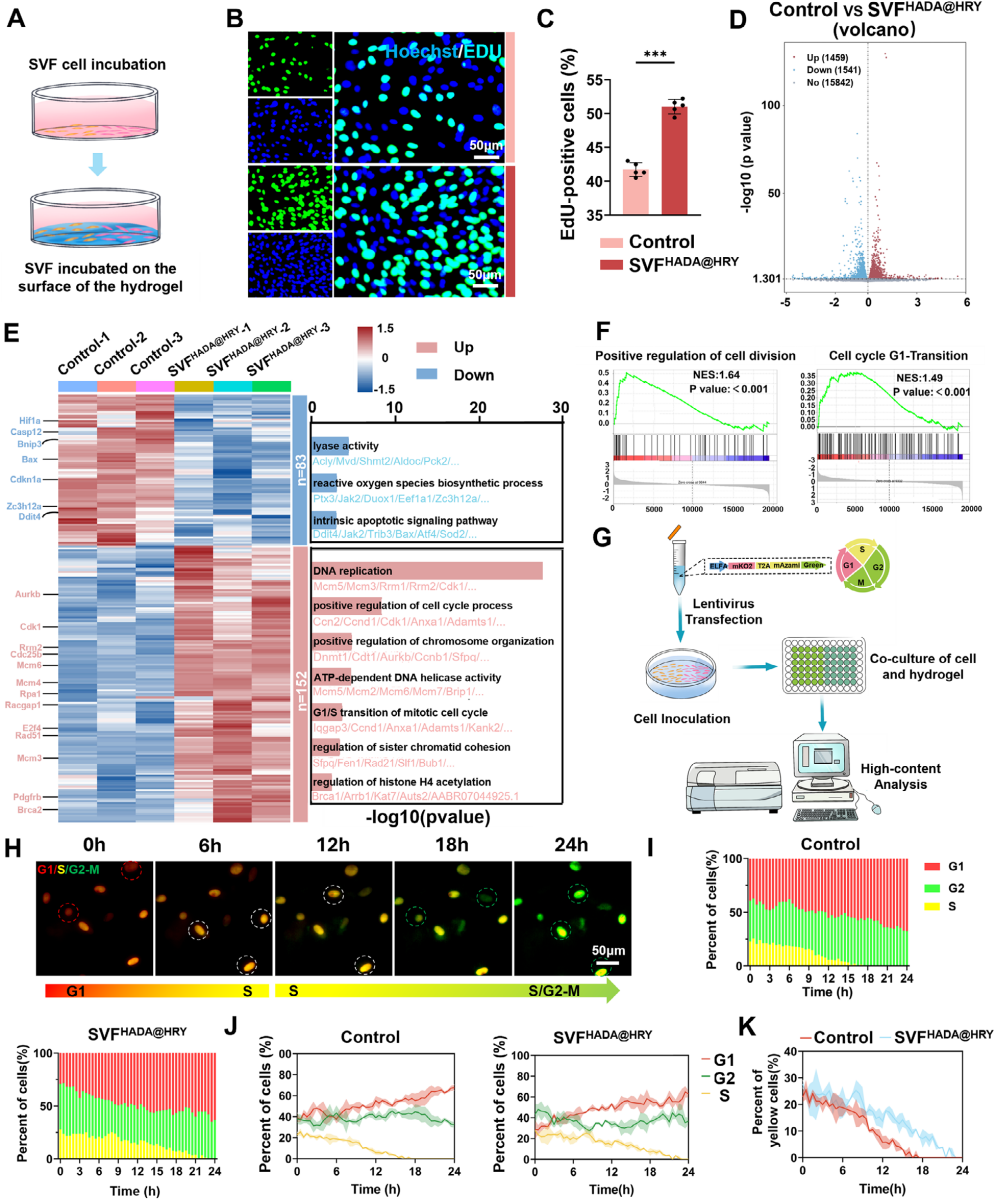
Effect of the HADA@HRY hydrogel on cell proliferation. A) Schematic diagram of the co‐culture of stromal vascular fraction (SVF) and HADA@HRY. B) Representative images of the EdU assay. C) Quantification of EdU‐positive cells in different treatment groups (n = 3). D) Volcano plot of differentially expressed genes (DEGs; red: upregulated; blue: downregulated; gray: nonsignificant). E) Heatmap of DEG expression levels (left) and functional enrichment and annotation (right) in SVF@HRY relative to control. F) Enriched pathways from the gene set enrichment analysis: Positive regulation of cell division and G1/S cell‐cycle transition. G) Protocol for real‐time cell cycle monitoring using the fluorescent ubiquitination‐based cell cycle indicator (FUCCI) system. H) Fluorescence time‐lapse imaging of cell cycle alterations in FUCCI‐expressing SVF cells post‐treatment. I) Percentage of SVF cells in different cell‐cycle phases across treatment groups. J) Dynamic changes in G1/S/G2‐M phase cell proportions under various treatments over time (n = 3). K) Dynamic changes in S‐phase proportions of SVF cells over time within different treatment groups (n = 3). Control: SVF cells; SVF^HADA@HRY^: SVF+ HADA@HRY hydrogel. All statistical data are represented as the mean ± standard deviation. No outlier exclusion or processing was performed during data analysis. Sample size (n) indicates independent biological replicates. Each experiment was performed with three biological replicates (n = 3). P values were calculated using unpaired two‐tailed Student's t test. ***P < 0.001.

Recognizing that enhanced SVF cellularity correlates with improved cartilage repair in OA [28], we investigated the proliferative effects of HADA@HRY on SVF cells. Abdominal fat‐derived SVF cells from Sprague‐Dawley (SD) rats were isolated and established as stable cultures (Figure S1A). HADA@HRY hydrogel‐mediated modulation of SVF cell proliferation was examined under co‐culture conditions (Figure 2A). To assess the proliferation rates, 5‐ethynyl‐2′‐deoxyuridine (EdU)‐positive SVF cells were identified via fluorescence microscopy. Quantitative analysis revealed that the EdU incorporation rates were elevated in the SVF^HADA@HRY^ group (51.01 ± 0.95%) compared to those in the control group (41.72 ± 0.90%) (Figure 2B, C).

To reveal the mechanistic basis of the HADA@HRY hydrogel‐driven cell fate modulation, RNA sequencing (RNA‐seq) was conducted to assess its impact on proliferation‐associated gene signatures in SVF cells. Inter‐sample Pearson correlation analysis revealed consistent clustering of biological triplicates per group (Figure S2A), validating experimental reproducibility. RNA‐seq revealed extensive reprogramming in SVF^HADA@HRY^, with 1,459 and 1,541 genes exhibiting elevated and suppressed expression levels, respectively, relative to the control group (Figure 2D). A comparative analysis between the control group and the SVF^HADA@HRY^ group revealed 235 significantly differentially expressed genes (DEGs), comprising 152 upregulated and 83 downregulated genes (Figure 2E). Gene Ontology (GO) analysis demonstrated that upregulated DEGs were predominantly enriched in DNA replication (e.g., *Orc6*, *Mcm2*) and positive regulation of chromosome organization (e.g., *Aurkb*, *Ccnb1*), whereas downregulated DEGs were significantly associated with the intrinsic apoptotic signaling pathway (e.g., *Bax*, *Cdkn1a*). Gene set enrichment analysis (GSEA) confirmed the activation of proliferation‐related pathways, particularly positive regulation of cell division and G1/S phase transition following HADA@HRY administration (Figure 2F; Figure S2B). These findings indicate that the HADA@HRY hydrogel stimulates cellular proliferation by accelerating cell‐cycle progression. High‐resolution spatiotemporal monitoring of single‐cell dynamics post‐hydrogel treatment was achieved using the fluorescence ubiquitination‐based cell cycle indicator (FUCCI) system [29, 30]. The red, yellow, and green fluorescent signals specifically marked cells in the G1, G1/S, and S/G2‐M phases, respectively (Figure 2G, H). The SVF^HADA@HRY^ group exhibited a significant accumulation of initially yellow‐fluorescent cells over 24 h, demonstrating a marked increase compared to those in other experimental groups (Figure 2I, J). Notably, a higher percentage of yellow‐labeled cells was observed in the SVF^HADA@HRY^ group (Figure 2K), suggesting that the hydrogel treatment facilitated cell‐cycle progression through the G1‐S checkpoint, thereby accelerating cell proliferation. To validate the anti‐apoptotic effects of HADA@HRY on SVF cells, we quantified terminal deoxynucleotidyl transferase‐mediated dUTP nick end labeling (TUNEL)‐positive cells in SVF populations. Assessment of apoptosis via TUNEL‐positive SVF cell enumeration confirmed HADA@HRY's anti‐apoptotic function, with the treated group showing significantly reduced apoptosis rates (10.44 ± 2.21% vs control 24.50 ± 3.56%; *P* < 0.05, Figure S3A, B). These results demonstrate that the HADA@HRY hydrogel promotes SVF cell proliferation by enhancing DNA replication, accelerating cell‐cycle progression, and suppressing apoptosis.

### 2.3 HADA@HRY Potentiated SVF Stemness for Cartilage/Bone Differentiation

**FIGURE 3:**
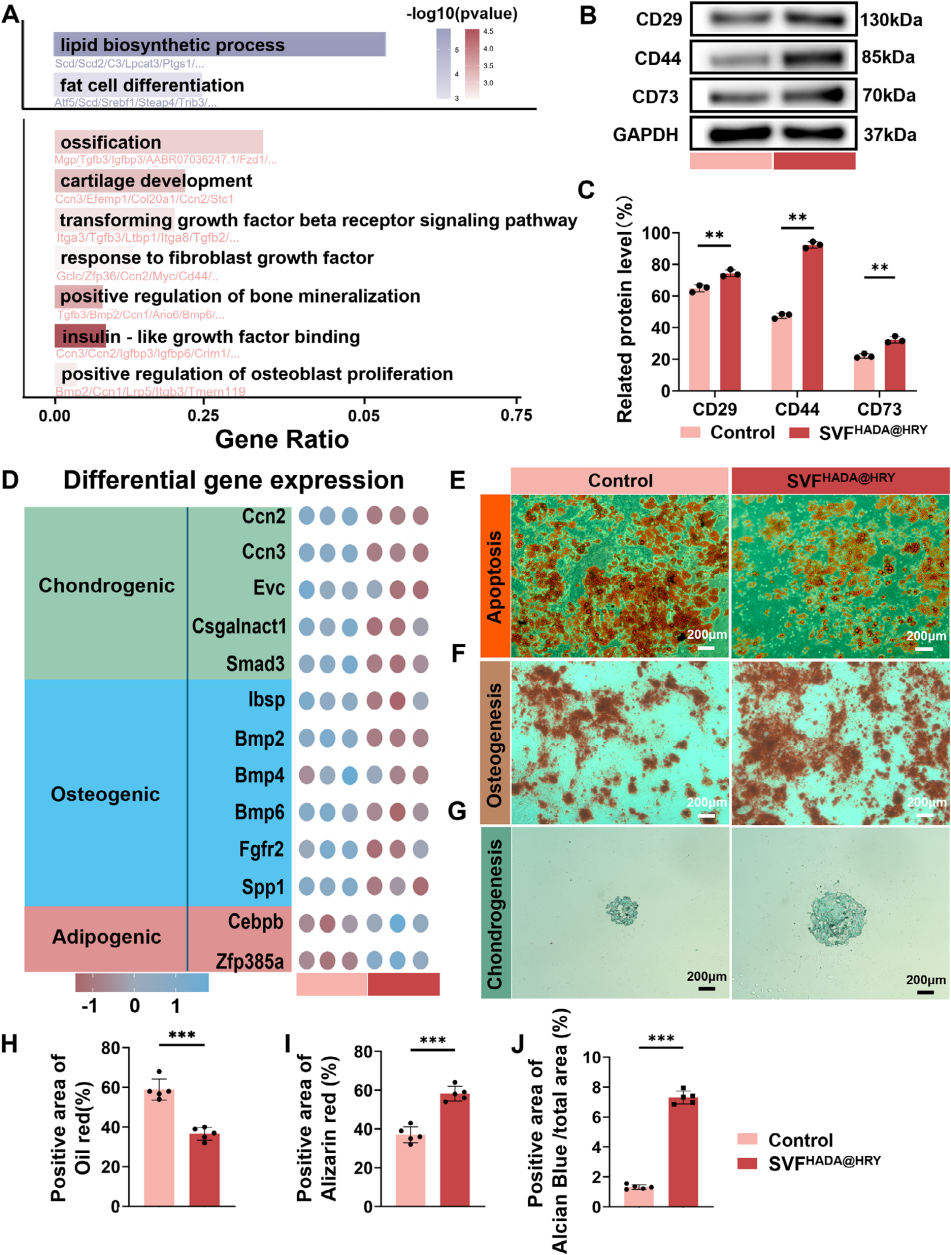
Properties of HADA@HRY‐enhanced stromal vascular fraction (SVF). A) RNA‐seq analysis: pathway regulation and gene enrichment (Control vs. SVF^HADA@HRY^). B) Expression levels of stemness proteins (CD29/CD44/CD73) were examined using western blot analysis (n = 3). C) Quantitative analysis of CD29/CD44/CD73 protein expression (n = 3). D) Chondrogenic, osteogenic, and adipogenic DEGs (Control vs. SVF^HADA@HRY^). E) Representative micrographs of Oil Red O staining. F) Percentage of Oil Red O‐positive area (n = 5). G) Representative images of Alizarin Red S staining. H) Percentage of Alizarin Red S‐positive area (n = 5). I) Fixation and Alcian blue staining of SVF‐derived pellets (Control vs. SVF^HADA@HRY^). J) Quantified percentage of Alcian blue‐positive area (n = 5). Control: SVF cells; SVF^HADA@HRY^: SVF+ HADA@HRY hydrogel. All statistical data are represented as the mean ± standard deviation. No outlier exclusion or processing was performed during data analysis. Sample size (n) indicates independent biological replicates. P values were calculated using unpaired two‐tailed Student's t test. ***P < 0.001.

SVF‐derived cells demonstrate positive expression of pluripotency‐associated markers and retain the capacity to differentiate into adipocytes, chondrocytes, or osteocytes upon lineage‐specific induction [31, 32]. GO analysis of HADA@HRY‐treated SVF cells revealed upregulated DEGs in chondrogenic‐and osteogenic‐related pathways, whereas downregulated DEGs were observed in adipogenic pathways (Figure 3A). Stem cell attributes are regulated by the expression of stemness genes [33]. Protein expression profiling through western blot analysis showed marked upregulation of stemness‐related surface markers (CD29, CD44, CD73) in HADA@HRY‐treated cells (Figure 3B, C). The data indicate that HADA@HRY hydrogel creates a favorable microenvironment for simultaneously maintaining SVF stem cell properties. Further gene expression analysis revealed upregulated chondrogenic genes (e.g., *Ccn2* and *Smad3*) as well as osteogenic genes (e.g., *Ibsp* and *Bmp4*) and downregulated adipogenic genes (Figure 3D). Quantitative reverse transcription PCR (qRT‐PCR) results revealed a marked increase in the expression of chondrogenic markers (*Sox9* and *Runx2*) and a concurrent decrease in adipogenic marker *Pparg* in SVF cells following HADA@HRY treatment (Figure S4A–C). We systematically evaluated the impact of SVF hydrogel on SVF cell differentiation along the osteogenic, chondrogenic, and adipogenic lineages. The results showed that the SVF^HADA@HRY^ group exhibited reduced Oil Red O‐positive areas (adipogenesis; Figure 3E, H), increased Alizarin Red‐positive areas (osteogenesis; Figure 3F, I), and enhanced Alcian Blue staining (chondrogenesis; Figure 3G, J). These outcomes suggest that HADA@HRY hydrogel intervention reinforces the pluripotency of stem cells, enhances the expression of genes associated with osteogenic and chondrogenic lineage‐specific differentiation, and enhances the capacity of SVF for chondrogenesis and osteogenesis, thereby offering a basis for reversing degenerative OA.

### 2.4 HADA@HRY Increased Ube2c+ ADSC Numbers With Superior Therapeutic Potential

**FIGURE 4:**
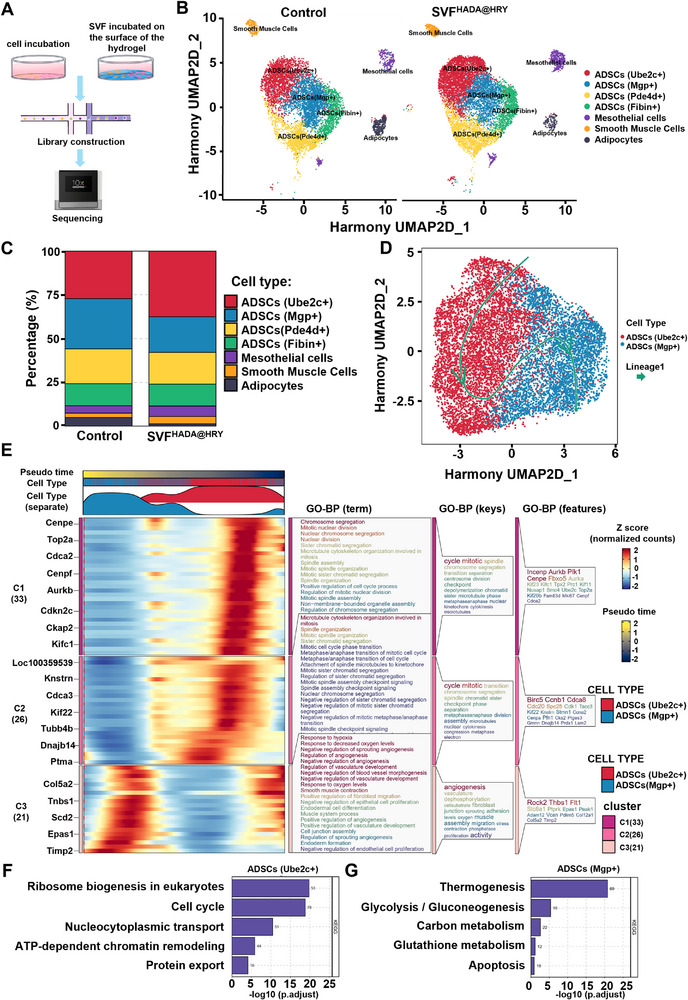
Single‐cell landscape in the SVF^HADA@HRY^ and control groups. A) Single‐cell RNA‐seq analysis workflow. B) Uniform manifold approximation and projection (UMAP) visualization of single‐cell data from SVF isolates (control: n = 8,498; SVF^HADA@HRY^ n = 11,319) identified seven transcriptionally distinct populations, as defined by canonical marker genes. C) Proportional distribution of cell types. D) Pseudotime trajectory analysis of adipose‐derived mesenchymal stem cells (ADSCs) via UMAP, revealing differentiation from Ube2c^+^ to Mgp^+^ states. E) Integration of heatmap visualization and Gene Ontology‐Biological Process (GO‐BP) enrichment analysis uncovered stage‐specific BPs in ADSC (Ube2c^+^ → Mgp^+^) differentiation. F‐G) Kyoto Encyclopedia of Genes and Genomes (KEGG) pathway enrichment analysis based on characteristic gene sets of ADSCs (Ube2c^+^) vs. ADSCs (Mgp^+^) cells. Control: SVF cells; SVF^HADA@HRY^: SVF cells + HADA@HRY hydrogel.

To identify systematic subpopulation changes in stem cell populations within the SVF, we explored single‐cell gene expression profiles from inguinal white adipose tissue (iWAT) of 6‐week‐old rats, followed by HADA@HRY intervention (Figure 4A). Through unsupervised clustering of single‐cell gene expression profiles, we identified seven major cell subtypes: Ube2c+ ADSCs, Mgp+ ADSCs, Pde4d+ ADSCs, Fibin+ ADSCs, mesothelial cells (MCs), smooth muscle cells (SMCs), and adipocytes (Figure 4B; Figure S5A, B). Subsequent proportional analysis of cellular populations revealed significant alterations in the Ube2c+ ADSC and Mgp+ ADSC subsets. Compared to the control group, the SVF^HADA@HRY^ group exhibited a 38.2% increase in Ube2c+ ADSCs and a 29.1% decrease in Mgp+ ADSCs (Figure 4C). Pseudotemporal ordering using the Slingshot algorithm delineated a unidirectional differentiation trajectory from Ube2c^+^ to Mgp^+^ ADSC subpopulations, with Ube2c^+^ cells occupying the trajectory root as progenitor cells (Figure 4D). GO term analysis showed that Ube2c+ADSCs were enriched in processes critical for stem cell maintenance, including DNA replication regulation and telomere maintenance (Figure 4E). In contrast, Mgp+ ADSCs were strongly linked to metabolic pathways, specifically those involved in cholesterol and ATP metabolic processes (Figure S6A, B). Kyoto Encyclopedia of Genes and Genomes (KEGG) pathway analysis confirmed distinct metabolic reprogramming: Ube2c^+^ ADSCs were enriched for cell cycle‐related pathways (“Cell cycle,” “ATP‐dependent chromatin remodeling”) (Figure 4F), whereas Mgp^+^ ADSCs showed signatures of energy metabolism (“Glycolysis/Gluconeogenesis,” “Carbon metabolism”) (Figure 4G). These findings demonstrate that HADA@HRY hydrogel treatment expands the stemness‐enriched Ube2c^+^ ADSCs subpopulation while reducing the less stemness‐competent Mgp^+^ ADSCs subset, highlighting a potential strategy to enhance SVF stemness.

### 2.5 HADA@HRY Regulated SVF Cell Senescence Via Foxm1‐Mediated Pathways

**FIGURE 5:**
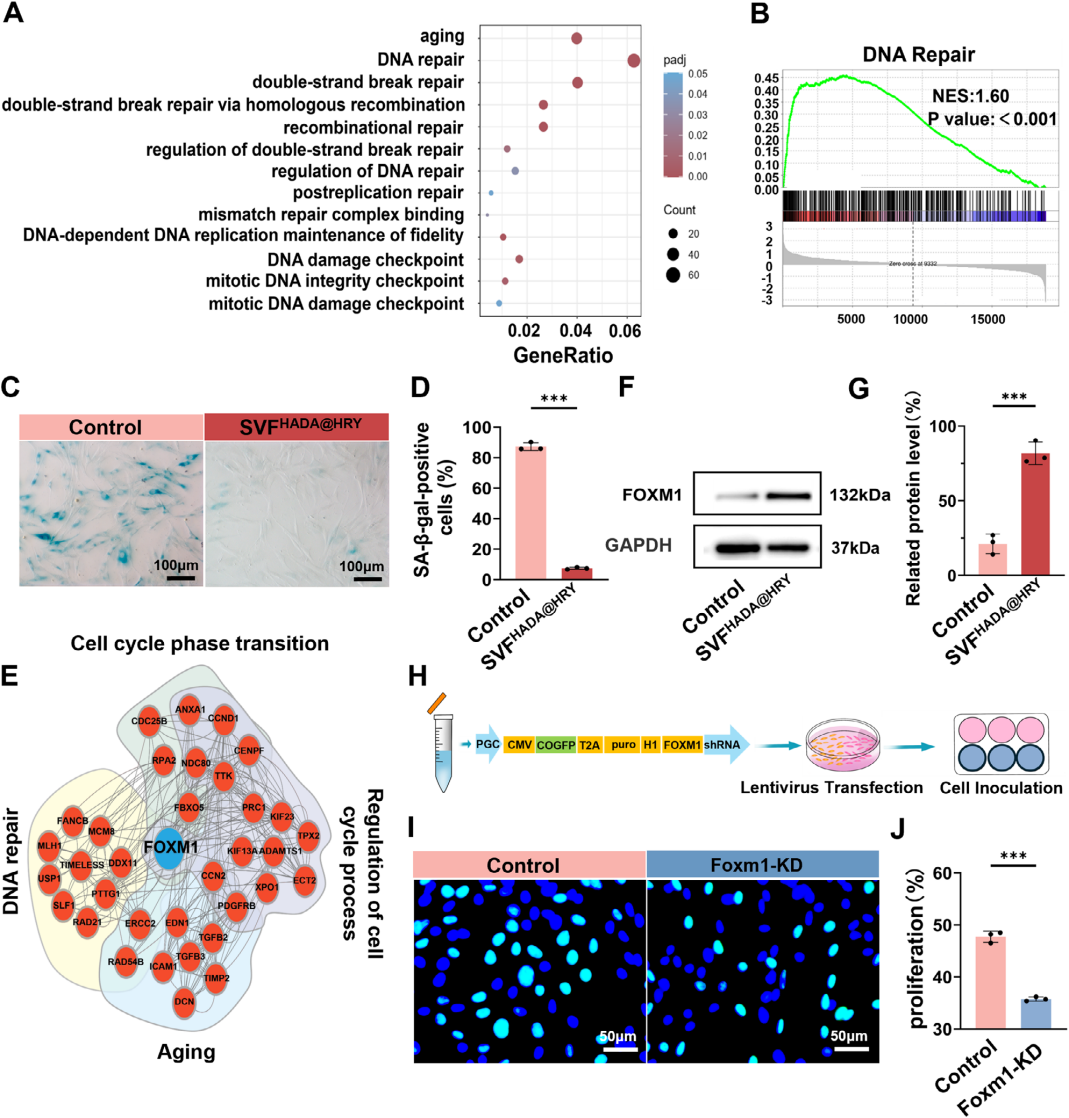
HADA@HRY regulates the expression of FOXM1. A) Scatter plot of enriched gene ontology pathways among the differentially expressed genes between the control group and SVF^HADA@HRY^. B) Gene set enrichment analysis shows upregulation of DNA repair pathways in HADA@HRY‐treated cells. C) Representative images of senescence‐associated β‐galactosidase (SA‐β‐gal) staining. D) Percentage of SA‐β‐gal‐positive cells (n = 3). E) Network analysis of FOXM1. F) Western blotting detection of FOXM1 (n = 3). G) Quantitative analysis of FOXM1 protein expression (n = 3). H) Lentiviral‐mediated delivery of sequence‐specific shRNA for stable Foxm1 knockdown model establishment. I) Representative images of EdU assay. J) Quantification of EdU‐positive cells in different groups. Control: SVF cells; SVF^HADA@HRY^: SVF+ HADA@HRY hydrogel. Foxm1‐KD: SVF cells + shRNA. For western blot analysis (panel G), band intensities were normalized to the GAPDH loading control. All statistical data are represented as the mean ± standard deviation. Sample size (n) indicates independent biological replicates. No outlier exclusion or processing was performed during data analysis. P values were calculated using unpaired two‐tailed Student's t test. ***P < 0.001.

Accumulation of DNA damage triggers cellular senescence in stem cells, leading to compromised self‐renewal capacity and skewed differentiation patterns [34]. GO analysis revealed upregulation of DEGs in pathways related to aging, DNA repair, double‐strand break repair via homologous recombination, and mitotic DNA integrity checkpoint following HADA@HRY intervention (Figure 5A). Notably, GSEA results established a mechanistic link between HADA@HRY intervention and enhanced DNA repair activity (Figure 5B). The pro‐repair effect of HADA@HRY on DNA damage was further corroborated by the quantification of γH2AX foci in SVF cells (Figure S7A). In comparison to the control, treatment with HADA@HRY resulted in a markedly decreased DNA damage rate (Figure S7B). SA‐β‐gal‐positive SVF cell counting confirmed the anti‐senescence effect of HADA@HRY (Figure 5C). Compared to the control group (87.3 ± 2.1%), the HADA@HRY‐treated group exhibited a significantly lower senescence rate (7.5 ± 0.6%; *P* < 0.05, Figure 5D). Western blot quantification further revealed a marked decrease in the levels of cyclin‐dependent kinase inhibitor 1A (CDKN1A) protein, a key senescence marker [35], following HADA@HRY treatment of SVF cells (Figure S8A, B).

Transcriptomic functional enrichment analysis of HADA@HRY‐treated SVF cells highlighted activation of cell‐cycle regulation, DNA repair mechanisms, and aging‐related pathways. Network‐based pathway analysis identified FOXM1 as a central hub governing stemness maintenance and senescence resistance (Figure 5E). Concomitantly, quantitative analysis confirmed elevated FOXM1 protein expression levels in HADA@HRY‐stimulated SVF cells (Figure 5F‐G). To functionally validate the regulatory role of Foxm1 in inhibiting SVF cell senescence and maintaining stemness, we established stable Foxm1‐knockdown (Foxm1‐KD) models using lentiviral delivery of sequence‐specific shRNAs (Figure 5H; Table S2). In SVF cells subjected to viral transfection, a significant decline in FOXM1 expression was observed (Figure S9A, B). In subsequent fluorescence microscopic quantification, the proportion of EdU‐positive SVF cells was reduced in Foxm1‐KD cells (35.4 ± 0.5%) relative to control cells (47.0 ± 1.0%; *P* < 0.05, Figure 5I–J). Quantitative analysis revealed that Foxm1‐deficient SVF cells contained significantly fewer yellow‐fluorescent‐labeled S‐phase cells than controls during the 24 h observation period (Figure S10A, B). Foxm1 downregulation also promoted senescence in SVF cells, as evidenced by significantly higher SA‐β‐gal positivity rates than untreated cells (Figure S11A, B). Collectively, these findings indicate that the HADA@HRY hydrogel exerts its regulatory effects on SVF cell function via Foxm1‐mediated pathways.

### 2.6 HADA@HRY Increased Chromatin Accessibility and Expression of Stemness‐Associated Genes

**FIGURE 6:**
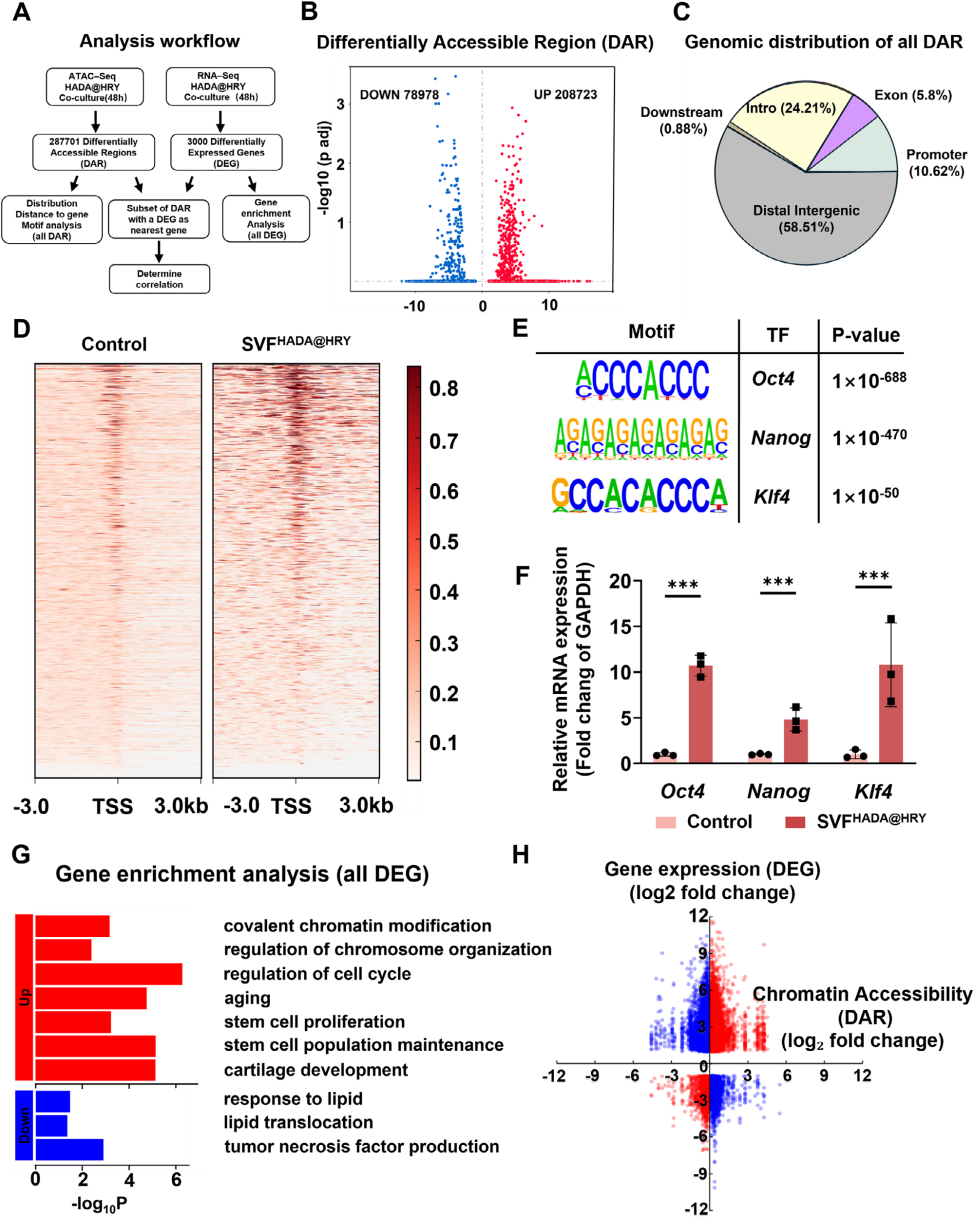
HADA@HRY enhances chromatin accessibility of stemness genes. A) Flowchart of experimental workflow and analytical procedures. B) Volcano plot of differentially accessible regions (DARs) in SVF cells after 48‐h HADA@HRY treatment, as identified by Assay for Transposase Accessible Chromatin using sequencing (ATAC‐seq) (red: increased accessibility; blue: decreased accessibility; gray: nonsignificant). C) Pie chart showing the genomic distribution of DAR peaks. D) Heatmap showing normalized ATAC‐seq signal intensity within ±3.0 kb windows centered on transcription start sites (TSS) (Control vs. SVF^HADA@HRY^; n = 3). E) Motifs enriched under inducible ATAC‐seq peaks at 48 h of HADA@HRY stimulation. F) qRT‐PCR analysis of *Oct4*, *Nanog*, and *Klf4* expression in SVF cells following 48‐h HADA@HRY treatment (n = 3). G) Metascape pathway analysis reveals biological processes associated with HADA@HRY‐mediated gene upregulation (red) and downregulation (blue). H) Scatter plot demonstrating a positive correlation between accessibility changes and expression of their nearest differentially expressed genes (*r* = 0.45, *P* < 0.0001, two‐tailed Pearson R test). Control: SVF cells; SVF^HADA@HRY^: SVF+ HADA@HRY hydrogel. For qRT‐PCR analysis, gene expression was normalized to GAPDH. Statistical significance was determined using two‐tailed Pearson's R test. All statistical data are represented as the mean ± standard deviation. Sample size (n) indicates independent biological replicates. No outlier exclusion or processing was performed during data analysis. values were calculated using unpaired two‐tailed Student's t test. **P < 0.01, and ***P < 0.001.

Having confirmed that Foxm1 could reinforce the stemness of ADSCs in SVF, we next performed an Assay for Transposase Accessible Chromatin using sequencing (ATAC‐seq) in physiological and HADA@HRY‐induced SVF cells to identify changes to open chromatin. Exposure to HADA@HRY increased the accessibility of 208,723 genomic regions, while 78,978 regions showed a loss of accessibility compared to SVF cultured in normal conditions (Figure 6A, B). Most differentially accessible regions localized to intronic/intergenic loci, while transcription start sites (TSS; ±3 kb) showed markedly enhanced accessibility, indicating transcriptional activation (Figure 6C, D). Furthermore, motif enrichment analysis of HADA@HRY‐induced ATAC‐seq peaks identified enriched binding motifs for pluripotency regulators, including *Oct4*, *Nanog*, and *Klf4* (*P* < 0.001; Figure 6E). Next, we performed quantitative reverse transcription PCR (qRT‐PCR) to assess the nuclear expression of these transcription factor families in HADA@HRY and under normal conditions. ATAC peak target genes were significantly upregulated in HADA@HRY treated cells (Figure S12A–C, Figure 6F; Table S1).

To correlate chromatin accessibility changes with gene expression alterations, we performed an integrative analysis of RNA‐seq and ATAC‐seq. When relating HADA@HRY‐mediated differentially accessible regions to their nearest gene, we found a positive correlation between chromatin accessibility changes and altered gene expression (*r* = 0.45, *P* < 0.0001) (Figure 6H). Upregulated genes associated with changes in proximal accessibility were enriched for pathways related to stem cell proliferation, cell‐cycle progression, and population maintenance. Conversely, suppressed genes were enriched in lipid response and translocation pathways, consistent with attenuated adipogenic commitment (Figure 6G). Taken together, our ATAC‐seq data suggest that as SVF cells respond to HADA@HRY, Foxm1 acts as a pioneer factor that promotes chromatin accessibility for other transcription factors.

### 2.7 HADA@HRY Hydrogel Enhanced the Efficacy of SVF in Osteoarthritis

**FIGURE 7:**
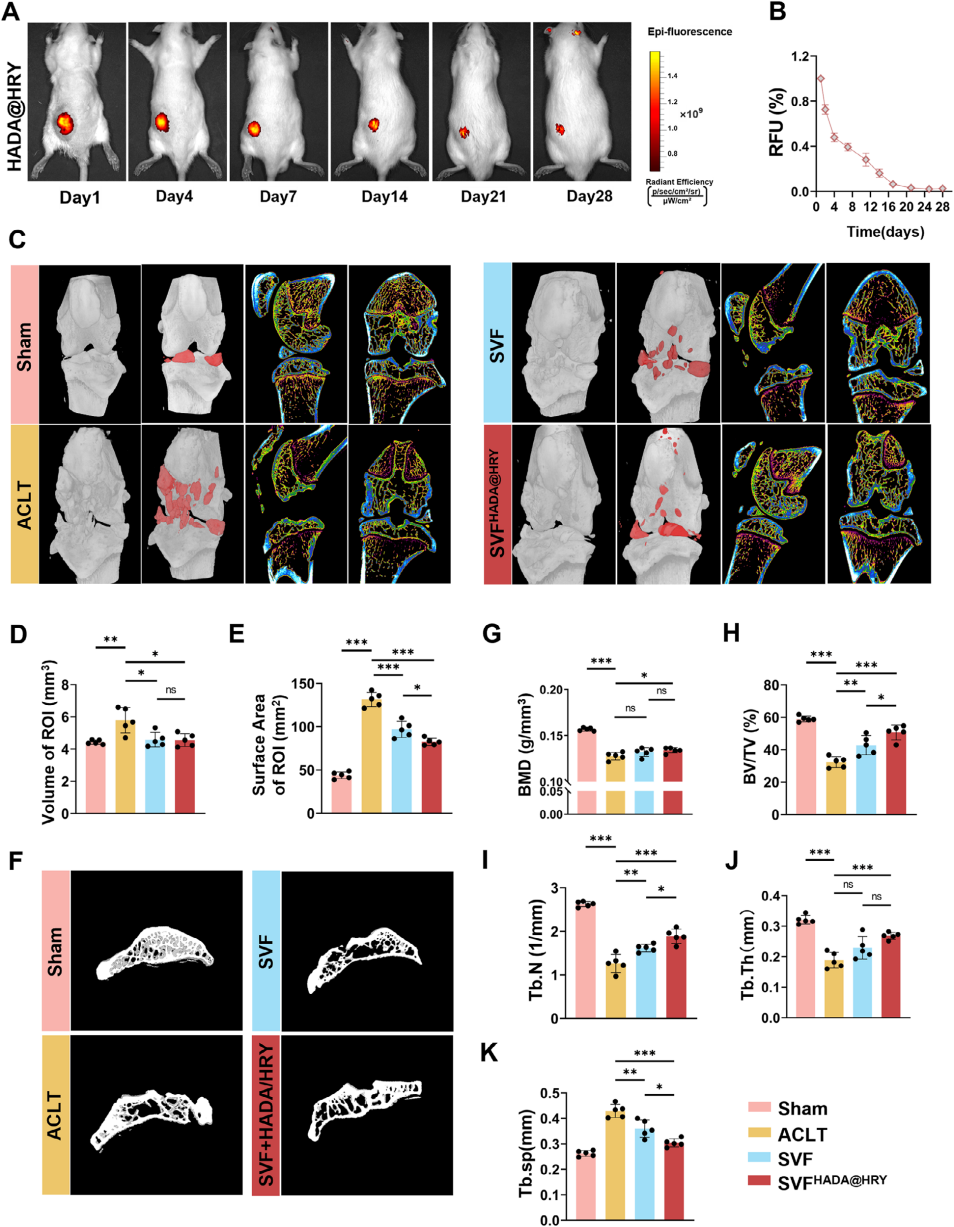
Combined stromal vascular fraction (SVF) and HADA@HRY hydrogel treatment effectively alleviates articular lesions in the knee joints following anterior cruciate ligament transection (ACLT). A,B) In vivo fluorescence imaging of hydrogels labeled with Cy5.5 (A) and quantification of fluorescence intensity (B; n = 3). C) Representative 3D (3D) micro‐CT scans of knee joints at 90 days after ACLT surgery. D, E) Micro‐CT‐based quantitative assessment of the surface area and the volume of the region of interest (ROI). F) Representative 3D micro‐CT images of sagittal visualizations of the medial compartment's subchondral bone at 90 days following sham operation or ACLT surgery. G) Quantitative micro‐CT investigation of the ratio of bone mineral density (BMD) in tibial subchondral bone and trabecular bone complex. H) Bone volume to total volume (BV/TV). I) Trabecular number (Tb.N). J) Trabecular thickness (Tb.Th). K) Trabecular separation (Tb.Sp). Sham = sham‐surgery. ACLT = ACLT‐surgery. SVF = ACLT‐surgery treated with SVF. SVF^HADA@HRY^ = ACLT‐surgery treated with SVF in conjunction with HADA@HRY hydrogel. Sample size (n) indicates independent biological replicates. No outlier exclusion or processing was performed during data analysis. All statistical data are represented as mean ± standard deviation. Statistical analyses were performed using one‐way ANOVA analysis of variance with Bonferroni's post‐hoc test. *P < 0.05,**P < 0.01, and ***P < 0.001.

To assess the in vivo stability of HADA@HRY hydrogels, Cyanine 5.5 monoacid (cy5.5), a near‐infrared fluorescent dye, was used. The hydrogels containing cy5.5 (0.001 mg/mL) were injected into the posterior dorsal subcutaneous tissue region of SD rats (n = 3), and their stability was monitored using an in vivo imaging system at distinct time intervals (1, 4, 7, 14, 21, 28 days) post‐implantation (Figure 7A). The HADA@HRY hydrogel retained a measurable fluorescence signal up to 21 days post‐implantation (Figure 7B). The results revealed that HADA@HRY hydrogel could persist in animals for an extended duration, enabling continuous facilitation of cell‐associated functions. At the endpoint (28 days), the harvested organs (heart, liver, spleen, lungs, and kidneys) were subjected to IVIS imaging, which identified the liver as the primary site of fluorescence retention. No signs of inflammation (e.g., macrophage aggregation) were detected in H&E‐stained liver and kidneys tissues, underscoring the hydrogel's biocompatibility (Figure S13A,B).

Next, we employed an anterior cruciate ligament transection (ACLT) rat model to evaluate the function of SVF^HADA@HRY^ in joint injury repair [36]. ACLT was conducted, and the rats were randomly assigned to four groups, with the sham‐operated rats serving as the controls. Intra‐articular SVF^HADA@HRY^ injection was performed on day 14 [37]. Subsequently, the rats were euthanized at 30 and 90 days post‐injection to represent the early phase of osteoarthritis featuring cartilage injury and the progressive phase exhibiting significant cartilage degradation, respectively [38, 39]. Joint tissues were collected for subsequent experimental procedures.

3D reconstruction from micro‐computed tomography (CT) scans indicated the presence of osteophytes in the ACLT group, with marked increases in both osteophyte volume and surface area (*P* < 0.01). As illustrated in Figure 7C, substantial roughness of the articular surface was notably extended in the ACLT group. At 30 days postoperatively, apparent degenerative changes were observed in the joints (Figure S14A), indicating severe injury to both articular cartilages. However, the SVF^HADA@HRY^ group showed significantly smaller volume of free osteophytes (4.56 ± 0.38 mm^3^) than the ACLT group (ACLT: 5.79 ± 0.82 mm^3^, *P* < 0.05, Figure S14B). The surface area of intra‐articular loose osteophytes was also reduced in the SVF^HADA@HRY^ group (47.01 ± 2.72 mm^2^) than that in the ACLT group (71.85 ± 9.07 mm^2^; *P* < 0.05, Figure S14C). By postoperative day 90, the therapeutic efficacy of SVF^HADA@HRY^ was further confirmed, exhibiting significantly smaller osteophyte volume (SVF^HADA@HRY^: 47%) than other groups (ACLT: 13.09 ± 1.08 mm^3^, SVF: 8.99 ± 1.03mm^3^; *P* < 0.05, Figure 7D) and reduced surface area of intra‐articular loose osteophytes (SVF^HADA@HRY^: 37.2%) compared to all other experimental groups (ACLT: 96.94 ± 9.21 mm^2^, SVF: 82.53 ± 4.25 mm^2^; *P* < 0.05, Figure 7E). In addition, the bone volume to total volume (BV/TV) showed increased bone mineral density following the application of SVF^HADA@HRY^ (Figure 7G, H).

OA induces progressive subchondral bone damage coupled with pathological osteophyte formation [40]. To comprehensively examine subchondral bone damage in ACLT rats, we tracked the dynamic changes in subchondral bone architecture during the development of OA. 3D micro‐CT imaging revealed marked structural alterations in the tibial subchondral bone of ACLT rats compared to sham controls (Figure 7F). At the 30‐day postoperative evaluation, micro‐CT analysis revealed that SVF^HADA@HRY^ therapy significantly enhanced the subchondral bone microarchitecture compared to both ACLT (*P* < 0.05) and SVF monotherapy (*P* < 0.05; Figure S14D–I). SVF^HADA@HRY^ demonstrated time‐dependent therapeutic effects, with significantly improved joint repair at 90 days compared to 30 days post‐treatment (*P* < 0.05). Specifically, the treatment group exhibited elevated levels of bone mineral density (+4.85% vs. +3.98%), BV/TV (+56.75% vs. +27.97%), Tb.N (+48.8% vs. +40.18%), and Tb.Th (+41.8% vs. +39.9%). In contrast, a reduction in Tb.Sp (−29.1% vs. −21.1%) was observed, suggesting enhanced trabecular connectivity and markedly improved bone quality (*P* < 0.05, Figure 7G‐K).

### 2.8 HADA@HRY Hydrogel Boosted the Therapeutic Efficacy of SVF in Joint Repair

**FIGURE 8:**
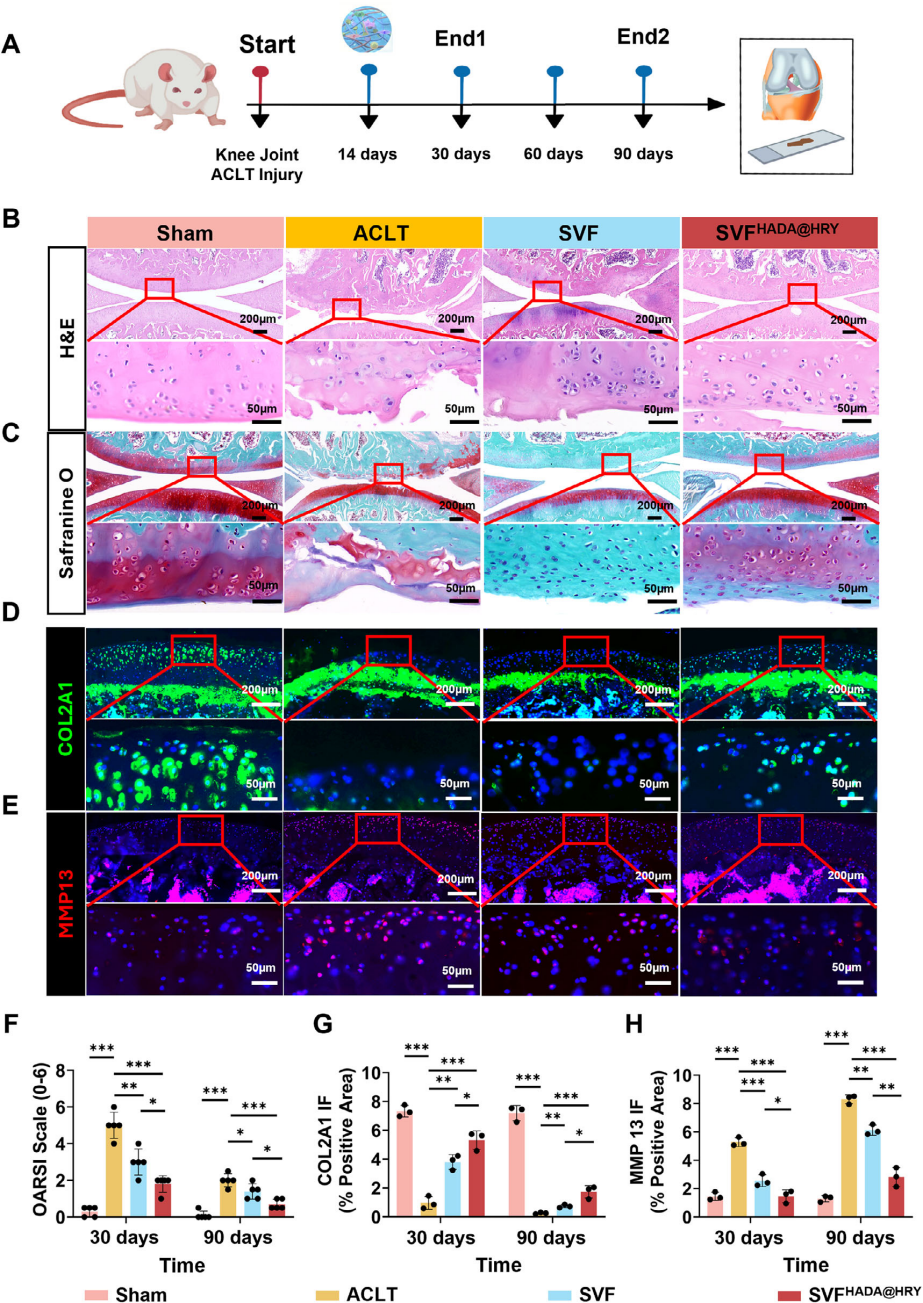
SVF and HADA@HRY hydrogel implantation preserves knee joint cartilage after ACLT. A) Schematic illustration of experimental design in vivo. B) Representative images of H&E‐stained sagittal sections at 90 days post‐surgery (n = 5). C) Safranin O‐fast green staining of joint cartilage area at 90 days post‐surgery (n = 5). D) Representative images of immunofluorescence staining of COL2A1 in knee cartilage (n = 3). E) Representative images of immunofluorescence staining of MMP‐13 in knee cartilage (n = 3). F) Quantitative analysis of Osteoarthritis Research Society International (OARSI) score of knee joints at 30 and 90 d postoperatively (n = 5). G) Quantification of COL2A1+ area of knee joints at 30/90 d post‐surgery. H) Quantification of MMP13+ area of the knee joints at 30/90 d post‐surgery. Sham = sham‐surgery. ACLT = ACLT‐surgery. SVF = ACLT‐surgery treated with SVF. SVF^HADA@HRY^ = ACLT‐surgery treated with SVF in conjunction with HADA@HRY hydrogel. Sample size (n) indicates independent biological replicates. All statistical data are represented as mean ± standard deviation. No outlier exclusion or processing was performed during data analysis. Statistical analyses were performed using one‐way ANOVA analysis of variance with Bonferroni's post‐hoc test. *P < 0.05,**P < 0.01, and ***P < 0.001.

The therapeutic efficacy of HADA@HRY hydrogel combined with SVF for OA was further validated by cartilage histology (Figure 8A). The OA group exhibited cartilage degradation, characterized by rough articular surfaces and degradation of glycosaminoglycans (GAGs). Hematoxylin‐eosin (H&E) and safranin O‐fast green staining showed cartilage regeneration and notable ECM reconstruction in the SVF and SVF^HADA@HRY^ groups (Figure 8B–C; Figure S15A, B). Joints receiving SVF injections exhibited enhanced structural restoration. SVF in conjunction with HADA@HRY hydrogel implantation resulted in significantly heightened COL2A1 staining, decreased MMP‐13 levels (Figure 8D, E; Figure S15C, D), and the most favorable OARSI score (Figure 8F–H). Time‐dependent efficacy was validated by improvements in OARSI scores (−60% vs. −50%), COL2A1 expression (+588.4% vs. +450.4%), and MMP‐13 reduction (−54.05% vs. −43.04%) at 90 days vs. 30 days, respectively, compared to the ACLT group.

## 3 Discussion

Stem‐cell implantation holds promise for cartilage regeneration in OA [41]. However, regenerative therapy remains challenging due to its limited efficacy in inflammatory and mechanically loaded environments [42]. In this study, we demonstrated that hydrogel‐encapsulated SVF implantation can effectively facilitate functional recovery in a rat model of OA. The hydrogel, crafted from HADA and a designed peptide, is injectable and tissue‐adherent and exhibits cell reprogramming capacity. When pitted against conventional hydrogels, it notably boosts the self‐renewal and directional differentiation of ADSCs within the SVF. These attributes collectively contribute to its superior regenerative performance in OA.

The fibroblast growth factor (FGF) family comprises a group of polypeptide growth factors ubiquitously expressed in multicellular organisms [43]. Consisting of 23 members (FGF1‐FGF23), these ligands mediate intricate signaling cascades through FGF‐FGFR complexes, orchestrating various aspects of development, homeostasis, and repair [44, 45]. As a multifunctional single‐chain growth factor of the FGF family, FGF2 serves as a critical modulator in cell fate determination throughout developmental processes [46]. At embryonic day 14.5 (E14.5), FGF2 is expressed in the cartilage condensations of the limbs, where it regulates chondrocyte proliferation within the growth plate [47, 48, 49]. Moreover, *FGF2* knockout mice exhibit reduced bone mass, alongside accelerated spontaneous and mechanically induced OA [50]. Emerging evidence demonstrates that FGF2 can promote the proliferation of MSCs through FGFR1/FRS2α‐mediated activation of AKT/ERK signaling pathways [51]. Additionally, FGF2 enhances the expression of the chondrogenic marker SOX9 in MSCs via the MAPK pathway, with all four FGFR subtypes potentially contributing to this activity [52]. These results demonstrate that FGF2 is an effective agent for enhancing MSC proliferation and chondrogenesis. Leveraging the pleiotropic properties of FGF2, we engineered RADA16‐I with an FGF2‐active peptide YRSRKYSSWYVALKR domain to enhance the synergy of the hydrogel.

Although physically entrapped growth factors impart bioactivity to hydrogels, their non‐covalent association leads to critical drawbacks, including protease vulnerability, uncontrolled release, and heterogeneous distribution, resulting in concentration gradients [53, 54, 55]. Conversely, covalent immobilization offers superior benefits through enhanced peptide stability and accurate spatial patterning, making it ideal for sustained stem‐cell niche maintenance in applications such as cartilage regeneration [56, 57]. Given the fundamental role of FGF2 in stem cell biology and the superior reliability of covalent conjugation strategies, we constructed the self‐assembling peptide HGF‐(RADA)_4_‐R‐YRSRKYSSWYVALKR. Its spontaneous organization with HADA through non‐covalent interactions yields the HADA@HRY hydrogel with a mechanically stable 3D architecture, specifically engineered for long‐term stem cell niche maintenance in demanding applications, such as osteochondral regeneration. In this system, HADA serves as a key multifunctional crosslinker and modifier. Its catechol groups not only enable covalent crosslinking through oxidation but also participate in dynamic physical interactions, thereby enhancing both the mechanical robustness and self‐healing capability of the hydrogel. Furthermore, HADA contributes to improved injectability and cytocompatibility, making it essential for constructing a versatile hydrogel platform suitable for cell delivery applications. Our results demonstrated that the HADA@HRY hydrogel effectively augmented SVF cell stemness, boosted proliferative capacity, and inhibited apoptotic pathways via sophisticated microenvironmental reprogramming. These effects mirror the well‐documented roles of FGF2 in stem cell maintenance and growth promotion, confirming that the designed FGF2‐mimetic peptide faithfully reproduces the biological activity of endogenous FGF2 in this hydrogel platform. Intriguingly, the hydrogel system successfully reversed the inherent adipogenic predisposition of SVF cells while markedly enhancing their chondrogenic and osteogenic differentiation potential through precise modulation of differentiation bias. This functional reprogramming confers significant therapeutic advantages for SVF‐mediated OA treatment.

The cell cycle comprises four sequential phases [58]. The G1 phase serves as the preparatory stage for DNA replication, characterized by extensive synthesis of RNA and proteins, and exhibits variable length. This is followed by the S phase, dedicated to the accurate duplication of DNA, thereby doubling the genetic content. Subsequently, the G2 phase functions as the final interphase checkpoint, involving the production of requisite proteins and inspection of DNA replication fidelity. The cycle culminates in the M phase (mitosis), which is notably the briefest stage [59, 60]. Cell proliferation reflects the net increase in cell number resulting from division, while the cell cycle constitutes the intricately regulated microscopic program that governs this process [61]. Our transcriptomic analysis showed that the hydrogel microenvironment potently induced upregulation of genes implicated in cell cycle progression, G1/S phase transition, DNA damage repair, and checkpoint regulation in SVF cells. Notably, these changes were not indicative of DNA damage‐induced cycle arrest; rather, they represented an adaptive augmentation of stem cell genomic stability and proliferative fidelity. Improved DNA damage repair efficiency, together with augmented checkpoint monitoring capability, allowed cells to timely clear potential genetic aberrations and sustain high‐fidelity DNA replication, consequently minimizing G1 phase accumulation caused by genomic stress [62]. Consequently, hydrogel treatment facilitated a more rapid and secure G1/S transition in SVF cells, leading to enhanced proliferative capacity while preserving genomic integrity. Collectively, these results demonstrate that the hydrogel establishes an ideal niche coupling accelerated cycle progression with reinforced genome maintenance, essential for SVF stemness, proliferation, and therapeutic safety.

Among stem cell‐based therapies, SVF demonstrates remarkable advantages in regenerative medicine due to its minimally invasive harvestability and multicellular synergistic effects [63]. SVF is a heterogeneous cell population derived from adipose tissue, comprising various cell types, including ADSCs, endothelial progenitor cells, macrophages, and fibroblasts. Preclinical studies have demonstrated that SVF can promote cartilage repair and regeneration through various mechanisms. For example, the ADSCs within SVF can differentiate into chondrocytes, contributing to the synthesis of ECM components in the cartilage. Additionally, SVF‐derived cells secrete various bioactive factors, including growth factors and cytokines, which can modulate the local microenvironment in the joint, reduce inflammation, and enhance the survival and function of endogenous chondrocytes. However, the ADSC component of SVF shows attenuated stem cell properties and limited expansion potential, and its predominant adipogenic commitment significantly restricts its application in OA [64]. In this study, the HADA@HRY hydrogel increased the proportion of high‐stemness Ube2c‐positive ADSCs while simultaneously reducing low‐stemness Mgp‐positive ADSCs in SVF. The differential response likely originates from intrinsic characteristics: Ube2c‐positive ADSCs maintain elevated FGFR expression and enhanced mechanosensitivity to hydrogel stimuli, while Mgp‐positive ADSCs show limited plasticity due to epigenetic constraints [65]. Notably, mesothelial and smooth muscle cells exhibited minimal changes in their proportions, while adipocytes decreased. This adipocyte depletion was putatively mediated by FGF2 via adipogenesis inhibition through the ERK/MAPK signaling pathway [66]. Furthermore, in our animal experiments, the combined treatment of HADA@HRY hydrogel with SVF demonstrated significantly enhanced synergistic therapeutic effects compared to SVF alone. Radiographic evaluation revealed a marked suppression of osteophyte formation, while histological analyses confirmed significant improvements in cartilage thickness, proteoglycan deposition, and chondrocyte alignment. Molecular analyses demonstrated upregulated COL2A1 accompanied by downregulation of MMP13, an inflammatory mediator linked to TNF‐α signaling [67]. Collectively, this combination therapy potently promoted cartilage regeneration while suppressing inflammation.

Genomic instability drives stem cell senescence and functional exhaustion [68]. Specifically, accumulated DNA damage and chromosomal abnormalities predispose stem cells to senescence or apoptosis, reducing their stemness and self‐renewal properties [69, 70, 71]. Our findings provide evidence supporting the “DNA repair‐stemness” positive feedback loop. RNA‐seq analysis revealed the upregulation of genes involved in DNA repair pathways in HADA@HRY‐treated SVF cells compared to controls. Moreover, ATAC‐seq profiling of the chromatin landscape revealed enhanced accessibility of regulatory elements associated with core pluripotency factors, such as *OCT4* and *Nanog*, corresponding to their elevated transcriptional activity. Functional validation in cell‐based assays confirmed that HADA@HRY hydrogel's anti‐senescence effects on SVF cells were mediated by Foxm1 activation. This cross‐hierarchical regulatory coordination is inherently linked to Foxm1's dual functionality [72, 73, 74]. It not only facilitates the assembly of DNA repair complexes but also drives 3D chromatin remodeling, thereby activating pluripotency networks [75]. Collectively, these findings demonstrate that HADA@HRY hydrogel orchestrates a Foxm1‐centric regulatory network integrating chromatin remodeling and stemness maintenance.

Here, we present a novel strategy to optimize SVF cell therapy for OA via HADA@HRY hydrogel. To the best of our knowledge, this is the first study to identify Foxm1 as a key transcription factor governing stemness maintenance in SVF cells, thereby providing a critical theoretical foundation for stem cell‐based therapies in degenerative diseases. Nevertheless, Foxm1‐regulated downstream targets need to be identified and its epigenetic regulatory networks need to be characterized in future studies. Additionally, in vivo tracking of SVF cell fate within articular microenvironments poses significant technical challenges. Addressing these critical questions highlights key avenues for future research.

## 4 Experimental Section

### 4.1 Self‐Assembling Peptide

The self‐assembling peptide HRY was custom‐synthesized and purified by Qiang Yao Biotechnology Co., Ltd. (Hubei, China). HA was sourced from Toronto Research Chemicals Inc. (Toronto, Canada). DA was purchased from J&K Scientific Ltd. (Beijing, China). The carbodiimide coupling reagents, 1‐(3‐dimethylaminopropyl)‐3‐ethylcarbodiimide hydrochloride (EDC) and N‐hydroxysuccinimide (NHS), were obtained from Meryer Chemical Technology Co. Ltd. (Shanghai, China).

### 4.2 Synthesis and Purification of HADA

The HADA conjugate was synthesized via a carbodiimide‐mediated coupling reaction. In brief, 758.6 mg of HA (2 mmol, based on the repeating unit) was dissolved in 120 mL of deionized water under continuous stirring at 35 °C in a nitrogen‐purged environment to prevent premature oxidation. Subsequently, NHS (690.5 mg, 6 mmol) and EDC (1.15 g, 6 mmol) were added to activate the carboxyl groups of HA, and the pH was carefully adjusted and maintained between 5.0 and 5.5 using dilute HCl or NaOH. After 15 min of activation, 189.6 mg of DA hydrochloride (1 mmol) was introduced into the reaction mixture. The reaction was completed in the dark at 35 °C for 24 h to prevent DA oxidation. Upon completion, the reaction mixture was transferred into a dialysis bag (MWCO: 3,500 Da) and dialyzed extensively against deionized water for 72 h, with frequent water changes. The dialyzed solution was frozen and lyophilized to obtain the dry HADA powder.

### 4.3 Preparation of HADA@HRY Hydrogels

Lyophilized HADA was first dissolved in deionized water to a final concentration of 2% (w/v) under constant stirring at 25 °C. Separately, the HRY peptide powder was weighed and gradually added to the HADA solution to obtain final peptide concentrations of 0%, 0.1%, 0.3%, and 0.6% (w/v). The mixture was stirred continuously until a clear and homogeneous solution was formed. Subsequently, the pH of each solution was carefully adjusted to 7.0 using 1 M Tris‐HCl buffer. The final solution was incubated at 37 °C for 30 min to allow complete gelation. The formed hydrogels were referred to as HADA@HRY hydrogel.

### 4.4 Isolation and Culture of SVF

All procedures involving animals were conducted according to the Institutional Animal Care and Use Committee (IACUC) guidelines and approved by the IACUC of Air Force Medical University (License No. IACUC‐20250024). Adipose tissue were harvested from the subcutaneous inguinal adipose tissue of SD rats (6–8 week) and sliced into small pieces. Dulbecco's Modified Eagle Medium (DMEM; Hyclone, USA) was added to facilitate cell activation. For tissue digestion, 0.1% type I collagenase (IC‐1108, InCellGene, BeiJing, China) was added, and the mixture was incubated at 37°C for 45 min and subsequently centrifuged at 203 × *g* for 10 min. The filtered precipitated cells were washed three times with normal saline and then resuspended. The cells were subsequently incubated in DMEM containing 10% fetal bovine serum (IC‐1901‐500, Incellgene, BeiJing, China) and 1% penicillin‐streptomycin antibiotics (0110AP‐100, Incellgene, BeiJing, China) at 37°C in a 5% CO_2_ atmosphere. The defined medium was replaced every 48 h, and the SVF was subcultured with trypsin‐EDTA (IC‐1709‐100, Incellgene, BeiJing, China) upon reaching 90% cell density. SVF within passages 2–4 was used for all experiments.

### 4.5 Microstructural Characterization of the Hydrogel

Cells were mixed with the hydrogel and cultured, followed by lyophilization for 48 h. The hydrogel was then sectioned to obtain cross‐sections. The sample surfaces were sputter‐coated with 10 nm gold. SEM was performed at 10 kV accelerating voltage (Hitachi, SU8010, Japan) across multiple magnifications to characterize both the hydrogel microarchitecture and attached cell morphology. Quantitative analysis of hydrogel pore diameter distribution was performed using the Nano Measure software (Nano Measure 1.2, Fudan University, Shanghai, China).

### 4.6 Single‐Cell RNA Sequencing (scRNA‐seq) and Analysis

scRNA‐seq was performed using the 10× Genomics platform (Chromium Next GEM Single Cell 3′ Reagent Kits v3.1) following the manufacturer's standard protocol. The computational analysis pipeline consisted of the following steps: The FASTQ files generated from 10× Genomics sequencing were processed using Cell Ranger to generate count matrices, which were subsequently read in HDF5 format and used to create Seurat objects with Seurat (v4.4.0). Cells were annotated with group information based on sample‐specific barcodes embedded in the cell identifiers. Batch effect correction was performed using the Integration_SCP function from the SCP package (v1.2.0), implementing the Harmony algorithm with a resolution parameter of 0.3 to integrate datasets across different samples. Quality control was performed using the RunCellQC function from the SCP package (v1.2.0), with cells filtered based on the following criteria: minimum UMI count >500, minimum gene count >300, and maximum mitochondrial plus ribosomal gene content <30%. Cluster results were subsequently visualized based on Uniform manifold approximation and projection (UMAP) dimensionality reduction. Cluster‐specific marker genes were identified using FindAllMarkers (avg_log2FC>0.5, p_adj<0.01) and visualized via DotPlot/FeaturePlot/VlnPlot. Differentially expressed genes were subjected to GO and KEGG pathway enrichment analysis using the RunEnrichment function from the SCP package (v1.2.0). For the selected cell subpopulations, the developmental trajectories were constructed using the Monocle2 (v2.24.0) package. The cellular state transition process was comprehensively characterized through pseudotime heatmaps and trajectory visualizations (depicting State, Pseudotime, and cell type annotations). For multi‐lineage trajectory inference, Slingshot (v2.8.0) was employed to reconstruct developmental trajectories by identifying principal curves in UMAP‐embedded space, automatically detecting main trunks and branching points.

### 4.7 OA Modeling and Objective Condition Assessment

Female SD rats (200‐240 g) were procured from the Laboratory Animal Center of Air Force Medical University. Rats were anesthetized by intraperitoneal administration of sodium pentobarbital. The OA model was established by performing ACLT on the right knee of rats. The surgical site was disinfected with povidone‐iodine solution postoperatively. A single intraperitoneal injection of penicillin at 10,000 U/kg was administered. The bedding material was replaced with fresh sterile substrate following the procedure. Sham‐operated rats were utilized as controls. Following surgical site disinfection, the rats were returned to their cages for standard housing with ad libitum access to food and water. The bedding material was replaced with fresh sterile substrate following the procedure. Intra‐articular drug administration was performed 14 days postoperatively under brief isoflurane anesthesia (2% in oxygen). The rats were randomly assigned to four groups for the injection of different preparations into the articular cavity: (1) saline, (2) SVF, (3) SVF+HADA@HRY, and (4) sham. The sham‐operated control group received equivalent saline injections following the same administration protocol as the experimental groups. In the SVF injection group, 100 µL of cell suspension containing 400,000 cells was injected. In the SVF + HADA@HRY group, 100 µL of the mixed preparation, which also contained 400,000 cells, was administered. Euthanasia was performed using an overdose of pentobarbital sodium at the designated time points of 30 and 90 days postoperatively, after which all rats were euthanized, and right knee joints were harvested for radiographic and histological evaluations.

### 4.8 Histological Analysis and IF

The knee joints of the euthanized rats were collected and immersed in 4% paraformaldehyde for a duration of 24 h. After rinsing for 24 h, the knee joints were transferred into an EDTA decalcification solution (Solarbio, China) for 2 mo. Following the decalcification process, dehydrated paraffin embedding was carried out. Using knee joint medial sagittal sections with a section thickness of 5 µm, cartilage sections were routinely deparaffinized and stained with H&E and Safranin‐O and Fast Green Staining for the assessment of cartilage morphology and GAG content. The histomorphology was visualized using a confocal microscope (Olympus FV3000). Additionally, joint status was assessed according to the OARSI scoring guidelines. For IF, 6 µm paraffin slices were dewaxed and treated with 3% H_2_O_2_ for 10 min at room temperature to eliminate endogenous peroxidase. For antigen repair, the joint portions were immersed in sodium citrate buffer, which was heated to 100°C for 8 min. Sections were blocked with 10% normal goat serum at room temperature for 2 h and incubated with the primary antibodies COL2A (1:100, ab34712, Abcam) and MMP13 (1:100, ab36861, Abcam) in a humid chamber at 4°C overnight (12 h). Following primary antibody incubation, samples were incubated with secondary antibodies at room temperature for 1 h, followed by DAPI staining for nuclear visualization. Confocal fluorescence microscopy was used for observation, and quantitative analysis was performed using ImageJ software (NIH, USA). The antibodies used are listed in Table S3.

### 4.9 Statistical Analysis

Data are presented as mean ± standard deviation, and all experiments were repeated at least three times. No data transformation or outlier exclusion was performed during analysis. Statistical analyses were performed using GraphPad Prism 8.0 software (GraphPad Software, Inc., USA). Comparisons between two groups were performed using an unpaired two‐tailed Student's t‐test. Comparisons among multiple groups were conducted using one‐way ANOVA followed by Bonferroni's post‐hoc test. Statistical significance was defined as *P < 0.05, **P < 0.01, and ***P < 0.001.

## Funding

This work was funded by the Shaanxi Provincial Key R&D Program (Grant No. 2023‐ZDLSF‐14), the National Natural Science Foundation of China (Grant Nos. 82572831, 32271417, 32071354, 92468101), and the Guangzhou Basic Research Program – Joint Funding Project of Municipal Government, Universities (Institutes), and Enterprises (Grant No. 2025A03J3216).

## Ethics Approval Statement

All procedures involving animals were conducted according to the Institutional Animal Care and Use Committee (IACUC) guidelines and were approved by the IACUC of Air Force Medical University (License No. IACUC‐20250024).

## Conflicts of Interest

The authors declare no conflicts of interest.

## Supporting information




**Supporting File**: advs75235‐sup‐0001‐SuppMat.docx.

## Data Availability

The data that support the findings of this study are available from the corresponding author upon reasonable request.
