# Supplementary material for: Self‐Assembling Hybrid Hydrogel Reprograms the Stromal Vascular Fraction to Treat Osteoarthritis
Source: Adv Sci (Weinh). 2026 Apr 20:e17297. Online ahead of print. doi: 10.1002/advs.202517297 (PMC13334670; doi:10.1002/advs.202517297)
Supplement: Supplementary file 1 — Supporting File: advs75235‐sup‐0001‐SuppMat.docx. [file ADVS-9999-e17297-s001.docx]

Supporting Information

**Self-Assembling Hybrid Hydrogel Reprograms the Stromal Vascular Fraction to Treat Osteoarthritis**

*Waifang Hou*^1, 3※^*, Yongfeng Chen*^1※^*^,^ Longyou Xiao*^2※^*, Wei Qi*^1※^*, Tianshu Du*^4^*, Qiang Sun*^1^*, Borui Xue*^3^*, Qinghe Zhao*^1^*, Pengfei Xie*^2^*, Zi Ye*^1^*, Fei Han*^1^*, Lingli Guo*^1^*, Yang Jiao*^5^*, Ranran Zhang*^5^*, Hong Jia*^1^*, Xiao Huang*^6^*, Jianwei Guo*^1^*, Peng Wang*^1^*, Huayi Wang*^1^*, Yuanrui Wang*^1^*, Wenjing Chen*^1^*, Haining Wu*^1^**, Liumin He*^2^ **, Dawei Zhang*^1^*

^1^Department of Orthopaedics, Xijing Hospital, The Fourth Military Medical University, Xi’an 710032, P.R. China

^2^Department of Spine Surgery, The Third Affiliated Hospital Sun Yat-Sen University Guangzhou 510630, China

^3^Air Force 986(th) Hospital, The Fourth Military Medical University, Xi'an, 710001, P.R. China

^4^Department of Nursing, Air Force Medical University, Xi’an, 710000, China

^5^Re-Stem Biotechnology Co. Ltd,2588 Wu zhong Road, Su zhou 215310, China

^6^Lintong Rehabilitation and Convalescent Centre of the Joint Logistics Support Force, Xi’an, 710600, China

*Email addresses: [zhangdw@fmmu.edu.cn](mailto:zhangdw@fmmu.edu.cn) (D. Zhang), [helm9@mail.sysu.edu.cn](mailto:helm9@mail.sysu.edu.cn) (L. He), [hnwu@fmmu.edu.cn](mailto:hnwu@fmmu.edu.cn) (H. Wu)

※W. Hou, Y. Chen, L. Xiao, and W. Qi contributed equally to this work.

Funding: This work was funded by the Shaanxi Provincial Key R&D Program (Grant No. 2023-ZDLSF-14), the National Natural Science Foundation of China (Grant Nos. 82572831, 32271417, 32071354, 92468101), and the Guangzhou Basic Research Program - Joint Funding Project of Municipal Government, Universities (Institutes), and Enterprises (Grant No. 2025A03J3216).

**Supplementary Experimental Section**

*UV-visible spectroscopy (UV-vs)*: The UV-Visible absorption spectra of HA, DA, and HADA were recorded using a UV-2450/2250 spectrophotometer (Shimadzu). A sample of 4 mg from each compound was dissolved in 1 mL of deionized water to ensure complete dissolution. The spectrophotometer was configured to scan across a wavelength range of 200 to 400 nm, with a step size of 0.5 nm for optimal resolution. The measurements were performed in automatic scanning mode to ensure consistency and accuracy throughout the spectral analysis.

*X-ray photoelectron spectrum (XPS)*: The lyophilized samples of both the HADA solution and HADA hydrogel were analyzed by X-ray photoelectron spectroscopy (XPS) using an ESCALAB 250 XPS system (Thermo Fisher Scientific, Waltham, MA, USA). The analysis was performed with a monochromatic Al-Kα X-ray source (1486.7 eV), which effectively excites the sample surface. High-resolution scans were acquired across a binding energy range of 0 to 1400 eV. Data processing and spectral analysis were conducted using the XPSPEAK41 software to interpret peak positions and identify chemical compositions.

*Rheological tests*: The mechanical properties of the hydrogels were evaluated using a Kinexus Pro rheometer (Malvern Instruments) with a 10 mm diameter parallel plate at 25°C. A 300 µL sample was placed between the plates, maintaining a 0.3 mm gap. Rheological testing was conducted in frequency sweep mode, spanning frequencies from 0.1 to 10 Hz, with a constant shear strain of 0.5%. The measurement process was repeated for multiple samples to ensure consistency.

*Injectability of hydrogel*: The injectability of the hydrogels was evaluated using a 1 mL syringe. The hydrogel was carefully loaded into the syringe, ensuring the absence of air bubbles. The hydrogel was then extruded at a consistent rate by applying controlled pressure to the plunger, simulating the injection process.

*Live/dead assay*: Cell viability was assessed using the Calcein-AM/PI Live/Dead Viability/Cytotoxicity Assay Kit (C2015M, Beyotime, China). Live cells exhibited green fluorescence, whereas dead cells showed red fluorescence. In this study, stromal vascular fraction (SVF) cells were mixed with HADA@HRY hydrogel and cultured for 24, 48, and 72 h, respectively. After incubation, the samples were washed three times with phosphate-buffered saline (PBS). Subsequently, staining solution was added, and the samples were incubated at 37 C for 30 min. Finally, the results were observed and recorded using a confocal microscope (Olympus FV3000).

*Proliferation assay*: Cell proliferation was evaluated using the 5-ethynyl-2'-deoxyuridine (EdU) incorporation assay (C0071S, Beyotime, China). Briefly, after 48 h of co-culture with HADA@HRY, SVF cells were incubated with EdU labeling solution for 2 h at 37 °C. The cells were then fixed with 4% paraformaldehyde (Servicebio, China) for 30 min and permeabilized with 0.3% Triton X-100 (ST1723-100, Beyotime, China) for 10 min. The incorporated EdU was detected using click reaction cocktail according to the manufacturer's protocol. Nuclei were counterstained with Hoechst 33342. Fluorescence images were captured using a confocal microscope (Olympus FV3000).

*Apoptosis assay*: Apoptosis was evaluated using a TUNEL assay kit (C1086, Beyotime, China). Briefly, after 48 h incubation with or without H₂O₂ treatment, SVF cells were fixed with 4% paraformaldehyde (Servicebio, China) for 30 min and permeabilized with permeabilization solution (P0097, Beyotime, China) for 5 min. Subsequently, cells were incubated with TUNEL reaction mixture for 1.5 h at 37 °C in the dark. Nuclei were counterstained with 4′,6-diamidino-2-phenylindole (DAPI; Beyotime, China), and images were captured using a fluorescence microscope (Olympus FV3000).

*Fluorescent ubiquitination-based cell cycle indicator (FUCCI) system*: The FUCCI system was employed to monitor cell-cycle progression in real-time.[1] A lentiviral vector (Genechem, China) overexpressing the FUCCI reporter genes (EF1α-mKO2-T2A-mAzami-Green) was constructed to enable cell cycle phase-specific expression during cell proliferation. Distinct fluorescent colors were used to identify different cell cycle phases: red fluorescence for the G1 phase, yellow for G1/S transition, and green fluorescence for the G2/M phase. According to the manufacturer's protocol, SVF cells were infected with the lentiviral vector for labeling. Following viral transduction, the labeled SVF cells were subjected to various experimental treatments. Cell cycle dynamics were then visualized and quantitatively analyzed using an Image Xpress Micro Confocal Imaging System (Molecular Devices).

*RNA sequencing and bioinformatics analysis*: Total RNA was extracted from the SVF cells of different treatment groups using TRIzol reagent. RNA-Seq libraries were prepared with the NEBNext Ultra™ Illumina RNA Library Prep Kit and subjected to paired-end sequencing. Raw sequencing data underwent quality control to generate “clean reads” for downstream analyses. For heatmap visualization, expression data were normalized using the “zero-to-one” scaling method via TBtools II software to enable accurate comparison of gene expression patterns. Subsequently, differentially expressed genes (DEGs) meeting the thresholds (P-value < 0.05, |Fold Change| ≥ 0) were functionally annotated through Metascape (https://metascape.org) for Gene Ontology (GO) enrichment and Kyoto Encyclopedia of Genes and Genomes (KEGG) pathway analyses to elucidate the underlying biological mechanisms.

*ATAC-sequencing*: Chromatin accessibility profiling was performed using an optimized assay for transposase-accessible chromatin using sequencing (ATAC-seq).[2,3] For each experimental group, three independent biological replicates of SVF cells were processed. Nuclei were isolated and resuspended in Tn5 transposase reaction mix (Illumina), followed by incubation at 37°C for 30 min to facilitate chromatin tagmentation. Post-tagmentation, equimolar concentrations of Adapter 1 and Adapter 2 were added, and the DNA fragments were amplified by PCR. The resulting libraries were purified using AMPure XP beads and quantified via Qubit fluorometry. Indexed libraries were subjected to cluster generation on a cBot system using the TruSeq PE Cluster Kit v3-cBot-HS, strictly following the manufacturer's protocol. Paired-end sequencing was subsequently performed on the Illumina NovaSeq™ X Plus platform with a 2×150 bp configuration.

*Quantitative Real-Time Polymerase Chain Reaction (qRT-PCR)*: Total RNA was extracted from snap-frozen SVF cells using TRIzol reagent (Ambion, USA) and purified with an RNA extraction kit (QIAGEN, Germany).[4] The extracted total RNA was subsequently reverse-transcribed into complementary DNA (cDNA) using the PrimeScript™ RT Master Mix (TAKARA, China). Quantitative real-time PCR analysis was conducted on a CFX96 system (Bio-Rad, Australia) under standard cycling conditions: initial denaturation at 95°C for 30 sec, followed by 40 cycles of 95°C for 5 sec and 60°C for 30 sec, with subsequent melt curve analysis (65-95°C). GAPDH served as the endogenous control for normalization, and relative gene expression was calculated using the 2-ΔΔCt method. All reactions included three technical replicates and no-template controls, with primer sequences detailed in Supplementary Table S1.

*Trilineage differentiation of SVF*: As previously described, the trilineage differentiation potentials of SVF were examined. In line with the manufacturer's instructions, all induction procedures were implemented. SVF cells were co-cultured with HADA@HRY, and then incubated in adipogenic (RAXMD-90031, OriCell), osteogenic (RAXMD-90021, OriCell), or chondrogenic (RAXMD-90041, OriCell) differentiation medium for 21 days in a 37 °C, 5% CO₂ incubator. The medium was replaced every 3 days throughout the induction period to maintain stable differentiation conditions. After induction, cells were washed with PBS and fixed with 4% paraformaldehyde at room temperature for 30 minutes, followed by staining with Oil Red O, Alizarin Red S, or Alcian Blue, respectively. Images of the differentiated SVF were captured and analyzed using confocal fluorescence microscopy.

*DNA Damage Induction and Detection*: SVF cells were seeded and grown to 60%–70% confluence. Subsequently, DNA damage was induced by exposing the cells to 125 ng/mL tumor necrosis factor-α (400-14-20UG, Thermo Fisher Scientific, USA) for 24 hours. Thereafter, SVF cells across all treatment groups were fixed in 4% paraformaldehyde for 15 minutes. Following PBS washes, permeabilization was performed with 0.3% Triton X-100 for 10 min, and blocking was carried out with 5% BSA for 1 h. The specimens were subsequently incubated with γ-H2AX (1:100, sc-517348, Santa Cruz Biotechnology) primary antibody overnight at 4°C, followed by a 1-h incubation with fluorescent secondary antibody at room temperature. Nuclear counterstaining with DAPI was performed prior to image acquisition via laser confocal microscopy.

*Cellular Senescence Induction and SA-β-Galactosidase Staining Assay*: SVF cells were treated with recombinant tumor necrosis factor-α (400-14-20UG, Thermo Fisher Scientific, USA) at a concentration of 30 ng/mL for 48 h to induce cellular senescence. SA-β-galactosidase staining was performed following the manufacturer's instructions (C0602, Beyotime, China). Briefly, for SVF cells of different groups cultured in 6-well plates, the culture medium was aspirated, and the cells were washed once with PBS (IC-2026-S, InCellGene, BeiJing, China). Subsequently, 1 mL of freshly prepared β-galactosidase staining fixative solution was added to each well, followed by fixation at room temperature for 15 min. After removing the fixative, cells were washed three times with PBS (3 min per wash). The staining reaction was carried out by incubating cells with the working solution at 37°C overnight. Fluorescent images were acquired using a confocal microscope (Olympus FV3000) with appropriate filters. Quantitative analysis of SA-β-gal-positive areas was performed using ImageJ software (NIH, USA).

*Table Knockdown of Target Genes*: To knockout *Foxm1*, we designed specific sgRNA sequences targeting the gene. These sgRNAs were then cloned into the CRISPR/Cas9 vector pSpCas9(BB)-2A-Puro (PX459) V2.0 (Genecarer, Xi'an, China), which carries both Cas9 and puromycin resistance genes. In this lentiviral system, the CMV immediate-early promoter controlled GFP and puromycin expression, while shRNA expression was regulated by the H1 promoter. HepG2 cells were transduced with three independent Foxm1-targeting shRNA constructs (shRNA-1, shRNA-2, and shRNA-3; Genecarer, Xi'an, China). A non-targeting negative control shRNA (NC) comprising scrambled sequences was included as control (Genecarer, Xi'an, China). The sequences are listed in Table S2.

*WB Assay*: The cells were passaged continuously until the ninth generation, reaching a confluency of 70–80% at each passage. The control group consisted of untreated cells. Cell lysates were prepared using radioimmunoprecipitation (RIPA) buffer to which 1% protease and 1% phosphatase inhibitors were added. A bicinchoninic acid (BCA) kit was utilized to assess the protein concentration (P0012, Beyotime, China). Proteins were separated by sodium dodecyl sulfate-polyacrylamide gel electrophoresis (SDS-PAGE) and then transferred onto a polyvinylidene difluoride (PVDF) membranes. The PVDF membranes were incubated for 1 h at 25 ° C in Tris-buffered saline with 0.1% Tween 20 (TBST) containing 5% BSA. Subsequently, the membranes were incubated overnight with primary antibodies at 4 °C. The membranes were washed three times with TBST (10 min each) and then incubated with secondary antibodies at room temperature for 2 h. Following three washes with TBST (5 min each), protein bands were visualized using enhanced chemiluminescence (ECL) substrate (ST673, Beyotime, China) and imaged using an imaging system (GE Healthcare, Stockholm, Sweden). All reactions were performed in triplicate (three technical replicates). The antibodies used are listed in Supplementary Table S3.

*In vivo biostability*: All procedures involving animals were conducted according to the Institutional Animal Care and Use Committee (IACUC) guidelines and were approved by the IACUC committee at Air Force Medical University (License No. IACUC-20250024). The hydrogels (0.3 mL) were directly injected into dorsal subcutaneous sites using a 1 mL sterile syringe. Each experimental group was tested with three implantations. Prior to the formation of the hydrogel, cyanine 5.5 monoacid (MCE, HY-D0924) was mixed with the hydrogel at a concentration of 0.001% (w/w). Fluorescence images of the cyanine - containing gels were captured at designated time points after surgery using a Visque InVivo Smart.

*Histological Analysis of Liver and Kidney Tissues:* Kidney tissues were collected from all experimental rats at the experimental endpoint (day 28 post-implantation), followed by routine paraffin embedding, sectioning (5 μm), and hematoxylin and eosin (H&E) staining. The histological morphology of renal tissues was observed using a confocal microscope (Olympus FV3000), and the renal tissue structure, including glomeruli and renal tubules, was carefully evaluated to assess potential toxic effects of the HADA@HRY system on renal function.

*Imaging examination*: Micro-CT scans of the joints were OBTAINED using the GE eXplore Locus SP system (Fairfield, USA) at 30 and 90 days postoperatively. The settings were as follows: resolution of 15 mm, voxel size of 27 mm, threshold of 1150, tube voltage of 80 kV, current of 80 mA, and exposure time of 3000 ms. Computed tomography (CT) scanning of Sprague-Dawley (SD) rat joints was performed to acquire consecutive tomographic images at 15 µM intervals, with all operations conducted by an experienced radiology technician possessing over five years of specialized expertise. Volume of interest (VOI) selection was carried out using Data Viewer software, followed by the acquisition of sagittal and coronal plane images of the articular region. The CTan software was used for the analysis of the joint bone quality, and the CTvox software was employed for the three-dimensional reconstruction of the images.

**Supplementary Results**

**
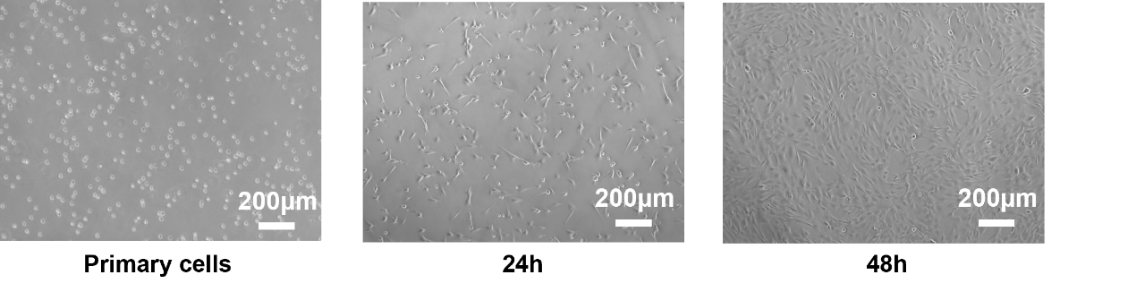
**

**Figure S1.** Primary SVF cell culture in vitro.

**
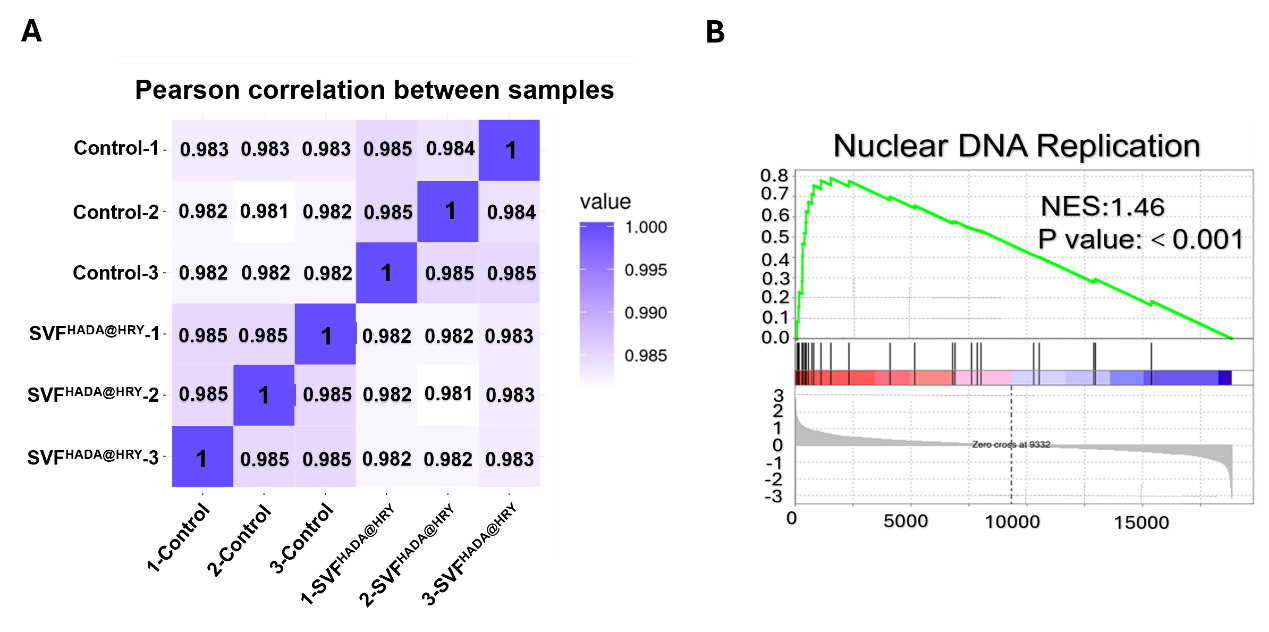
**

**Figure S2.** A) RNA-seq correlation analysis in HADA@HRY-treated SVF cells. B) Gene Set Enrichment Analysis (GSEA) demonstrated significant enrichment of nuclear DNA replication pathways following HADA@HRY administration.


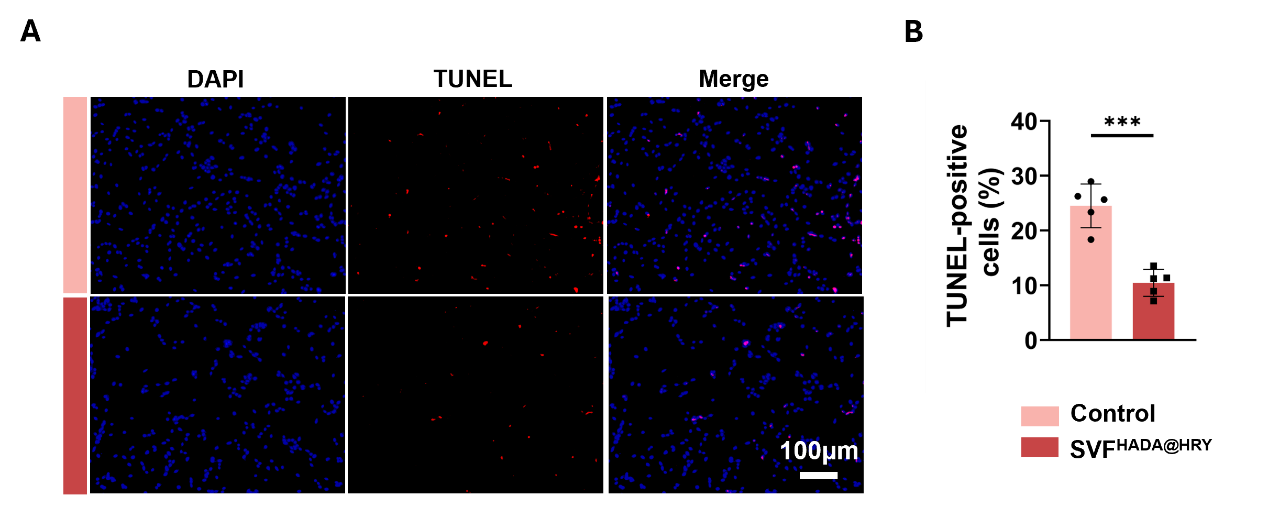
**Figure S3.** A) Apoptosis evaluation of SVF cells. B) Statistical analysis of the TUNEL+ cells in different groups (n = 5). Control: SVF cells; SVF^HADA@HRY^: SVF+ HADA@HRY hydrogel. All statistical data are represented as the mean ± standard deviation. P values were calculated using unpaired two-tailed Student’s t test. ***P < 0.001.

**
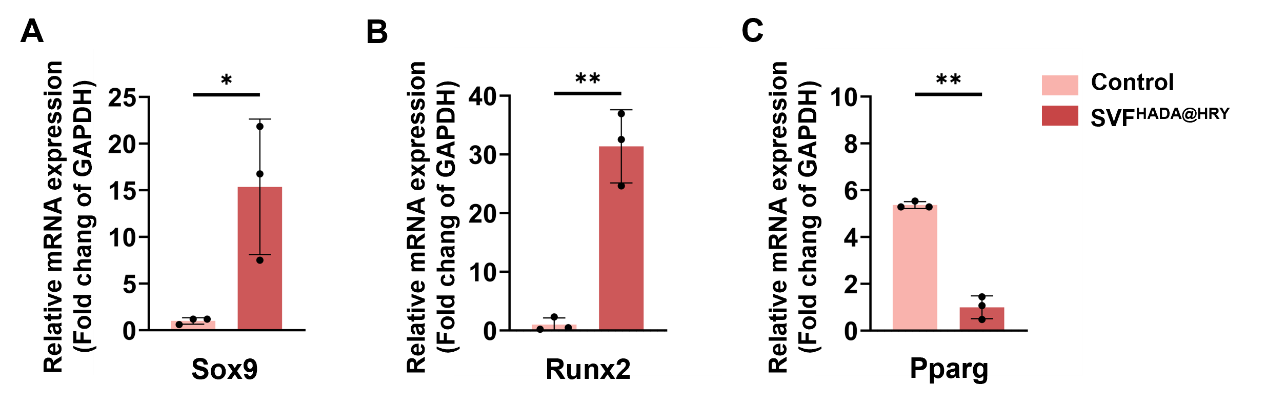
**

**Figure S4.** A-C) qRT-PCR analysis of *Sox9*, *Runx2*, and *Pparg* expression in SVF cells following HADA@HRY treatment (n = 3). Control: SVF cells; SVF^HADA@HRY^: SVF+ HADA@HRY hydrogel. All statistical data are represented as the mean ± standard deviation. P values were calculated using unpaired two-tailed Student’s t test. *P < 0.05. **P < 0.01.

**
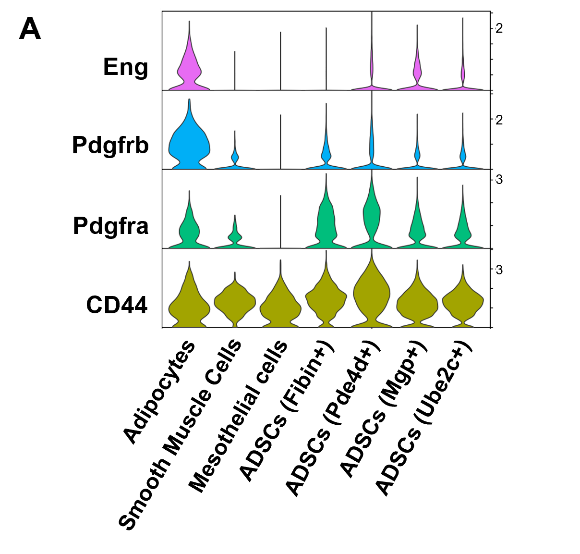
**

**
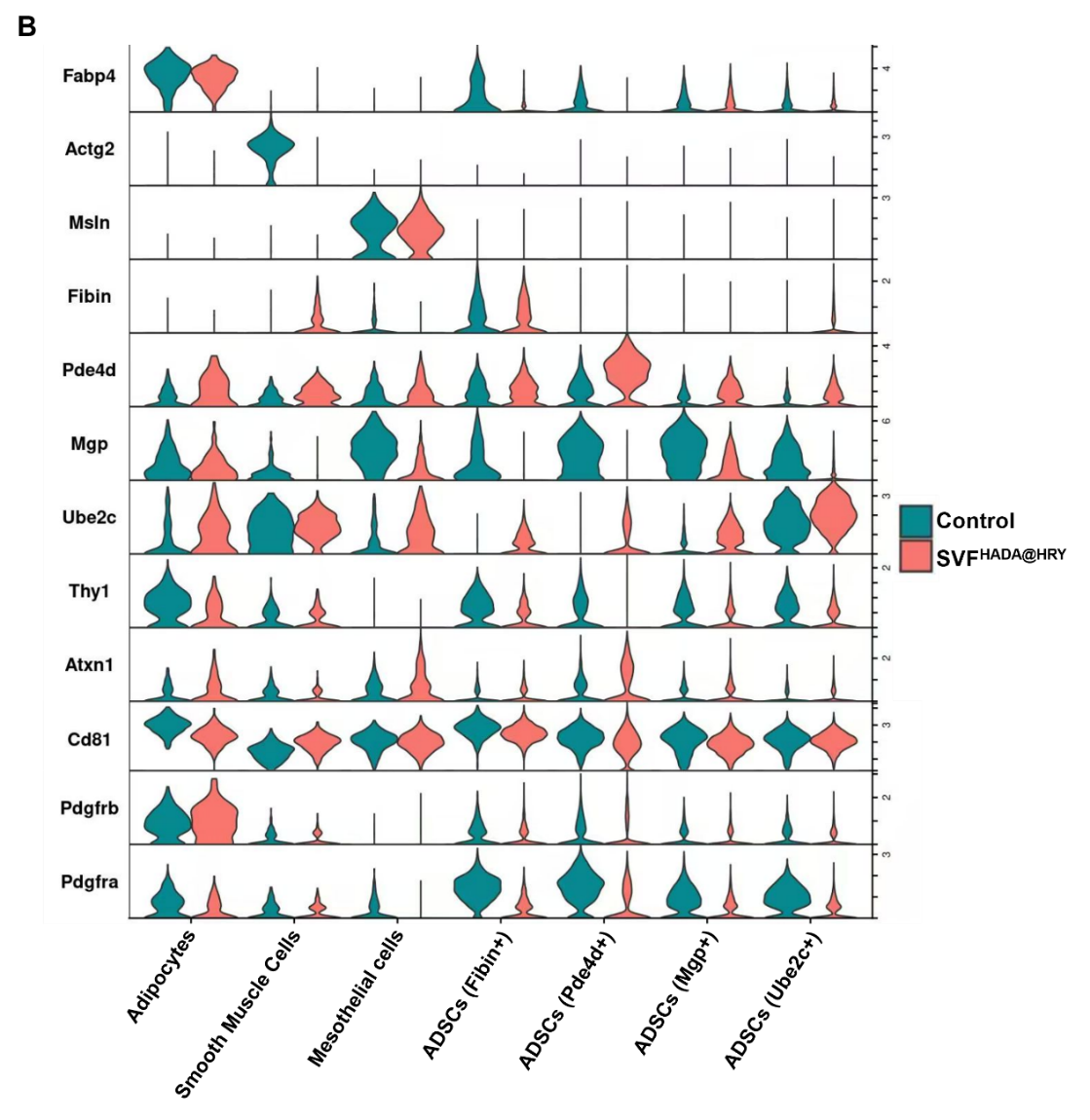
**

**Figure S5.** Mesenchymal stem cell and preadipocyte marker gene expression profiles across cell groups from adult iWAT. A) Expression of selected genes from the literature (mesenchymal stem cell markers), and cluster-specific marker genes. B) Violin plots display the mean expression density and variance of selected genes between the control and SVF^HADA@HRY^ groups.

**
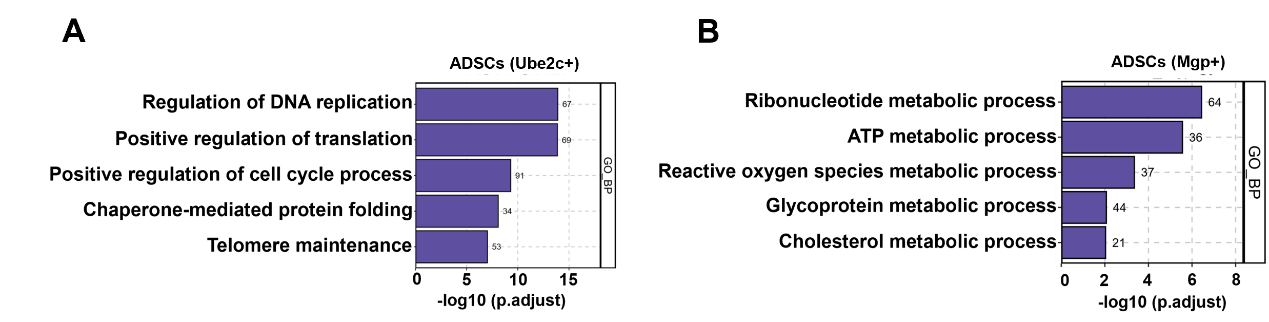
Figure S6.** A) Gene Ontology (GO) term enrichment analysis of Ube2c^+^ ADSC specific genes. B) GO term enrichment analysis of Mgp^+^ ADSC specific genes.

**
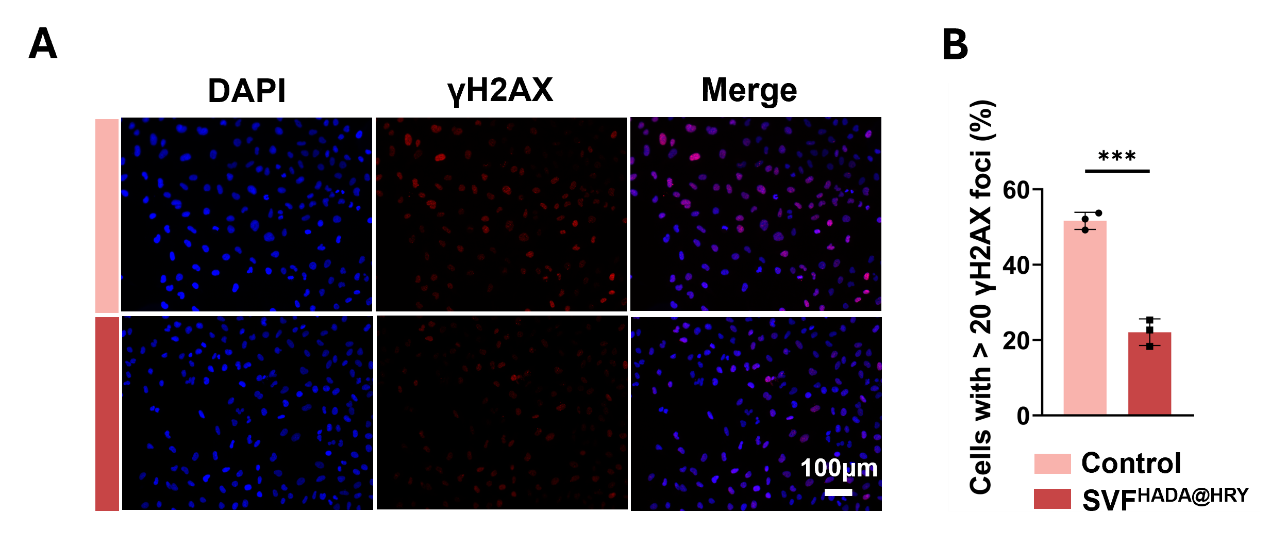
**

**Figure S7.** A) Representative images of γH2AX immunofluorescence staining. (n = 3). B) Quantitative analysis of the percentage of cells with >20 γH2AX foci per cell in different treatment groups. Scale bar: 100 μm. (n = 3). Control: SVF cells; SVF^HADA@HRY^: SVF+ HADA@HRY hydrogel. All statistical data are represented as the mean ± standard deviation. P values were calculated using unpaired two-tailed Student’s t test. ***P < 0.001.

**
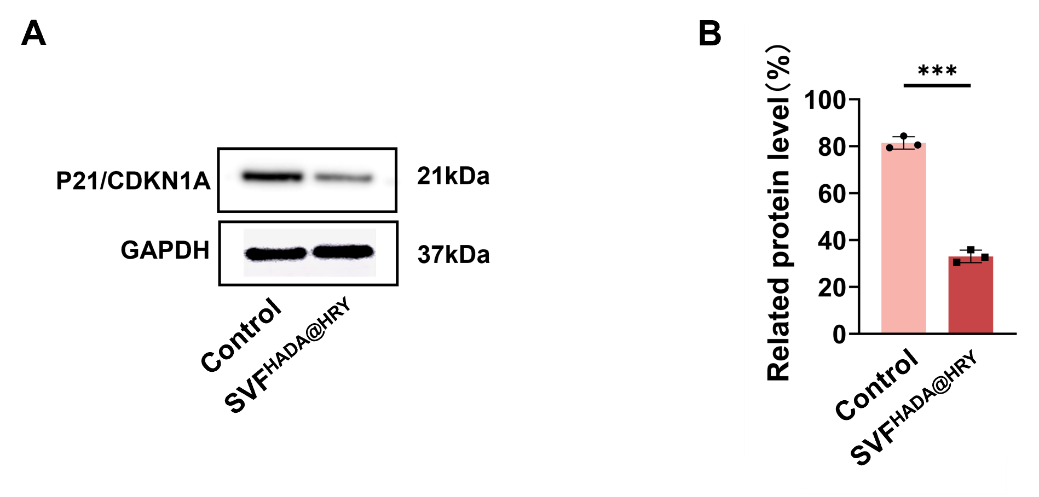
**

**Figure S8.** A) The expression levels of the aging-related protein cyclin-dependent kinase inhibitor 1A (CDKN1A) were determined by western blotting analysis (n = 3). B) Quantitative analysis of CDKN1A protein expression (n = 3).Control: SVF cells; SVF^HADA@HRY^: SVF+ HADA@HRY hydrogel. All statistical data are represented as the mean ± standard deviation. P values were calculated using unpaired two-tailed Student’s t test. ***P < 0.001.

**
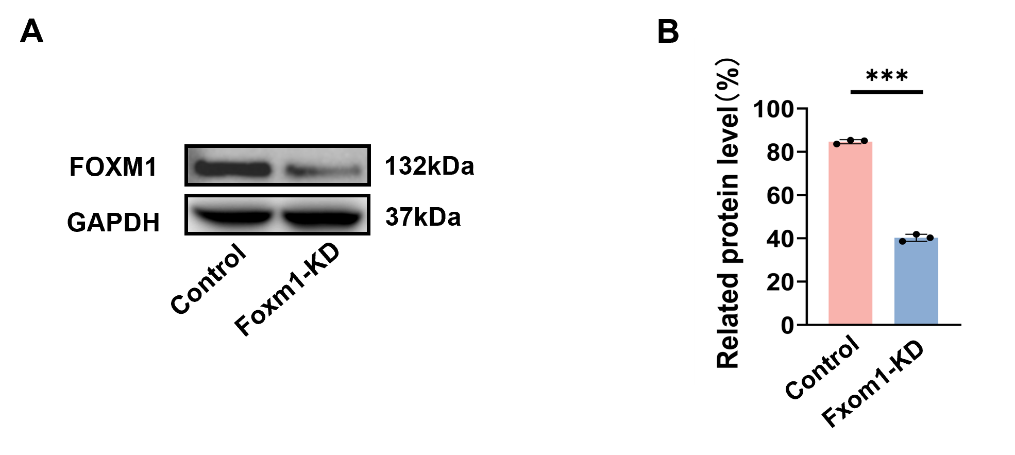
**

**Figure S9.** A)Western blot analysis of forkhead box M1 (FOXM1) expression in control cells and Foxm1-knockdown (Foxm1-KD) cells (n = 3). B) Quantitative analysis of FOXM1 protein expression (n = 3). Control: SVF cells; Foxm1-KD: SVF cells + shRNA. All statistical data are represented as the mean ± standard deviation. P values were calculated using unpaired two-tailed Student’s t test. ****P* < 0.001.

**
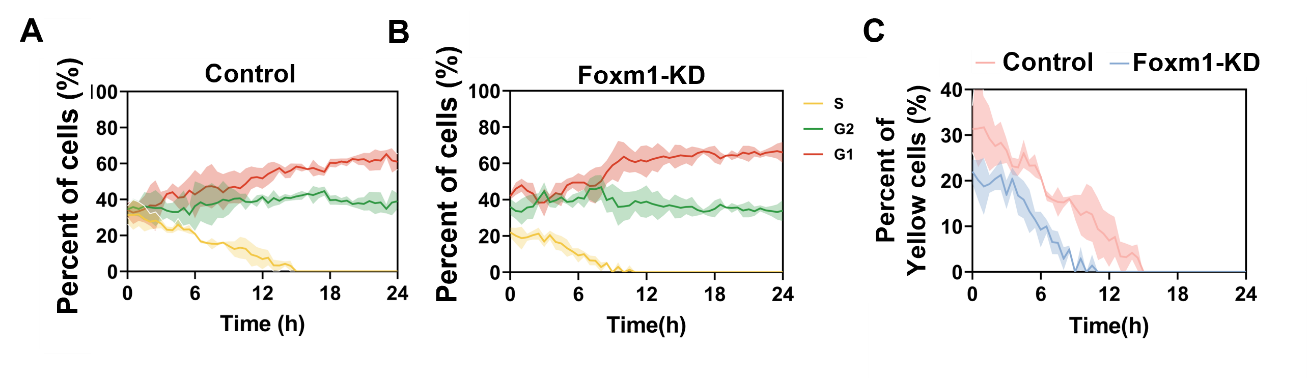
Figure S10.** A) Dynamic changes in G1/S/G2-M phase cell proportions in control groups over time (24 h; n = 3); B) Dynamic changes in G1/S/G2-M phase cell proportions in Foxm1-KD groups over time (24 h; n = 3); C) Dynamic changes in S-phase proportions of SVF cells over time within different treatment groups (24 h; n = 3)

**
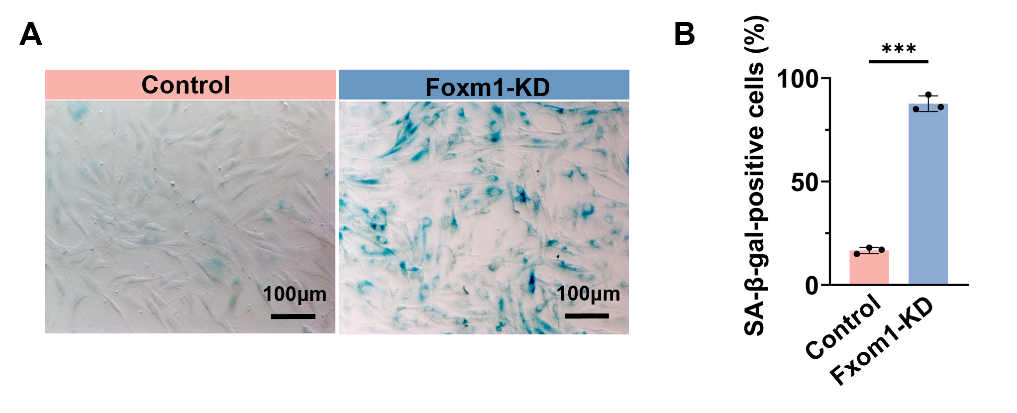
**

**Figure S11.** A) SA-β-gal staining of the control and Foxm1-KD groups. B) Percentage of SA-β-gal-positive cells (n = 3). Control: SVF cells; Foxm1-KD: SVF cells + shRNA. All statistical data are represented as the mean ± standard deviation. P values were calculated using unpaired two-tailed Student’s t test. ****P* < 0.001.

**
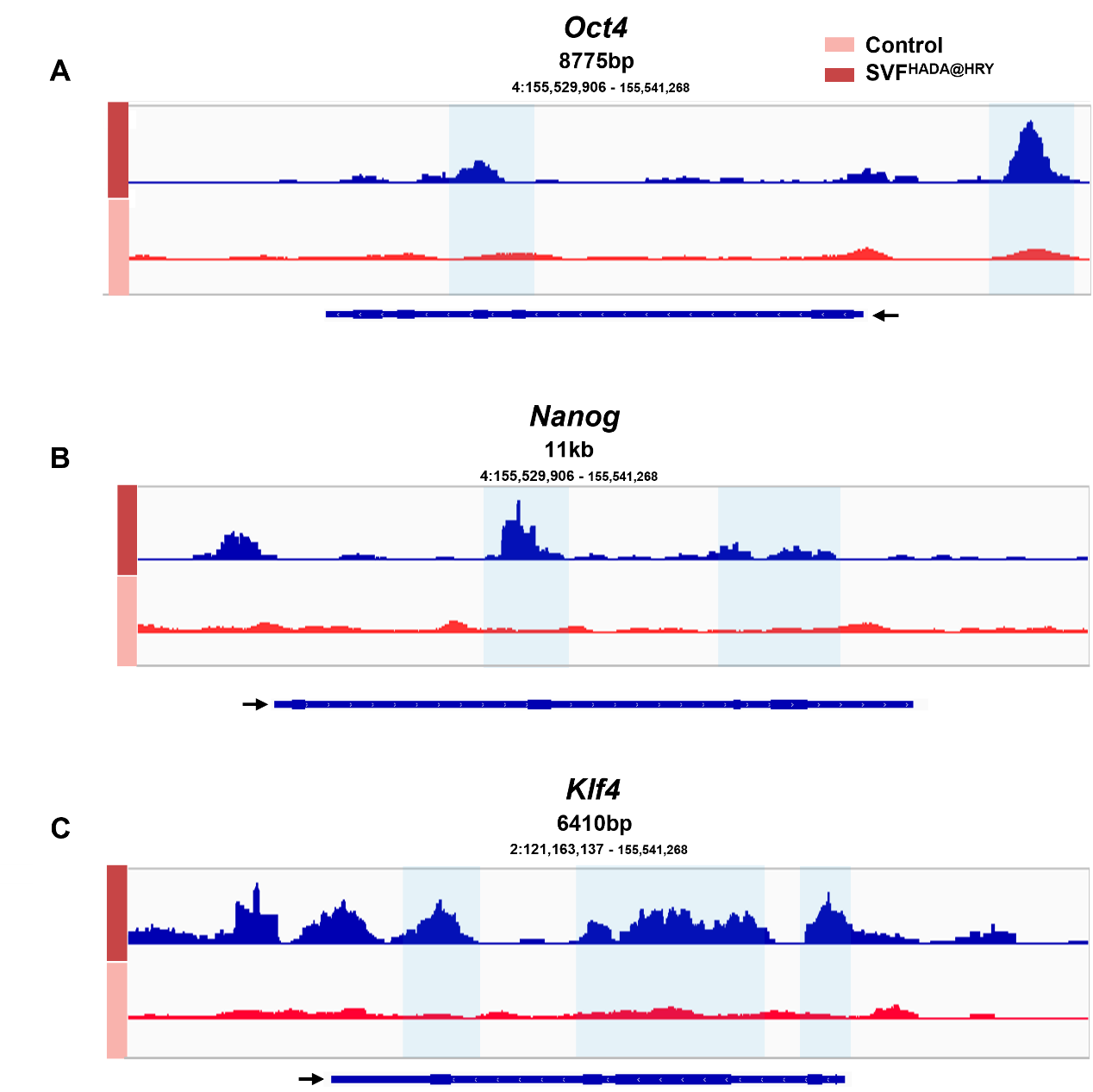
**

**Figure S12.** A-C) Gene expression profile of ATAC-seq signal intensity for *Nanog*, *Oct4*, and *Klf4* visualized in the genome browser.

**
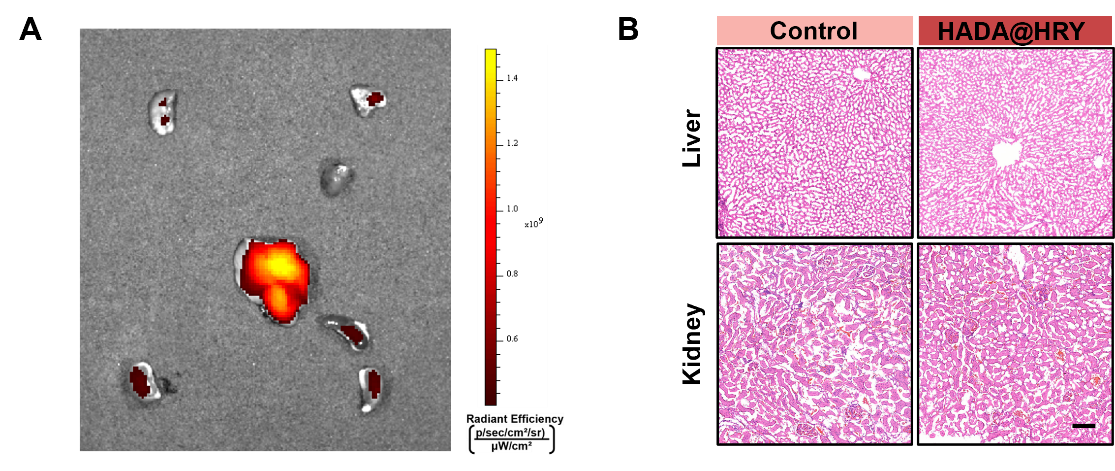
**

**Figure S13.** A) IVIS images of rat heart, liver, spleen, lung, and kidney. B) H&E-stained liver and kidney tissue.

**
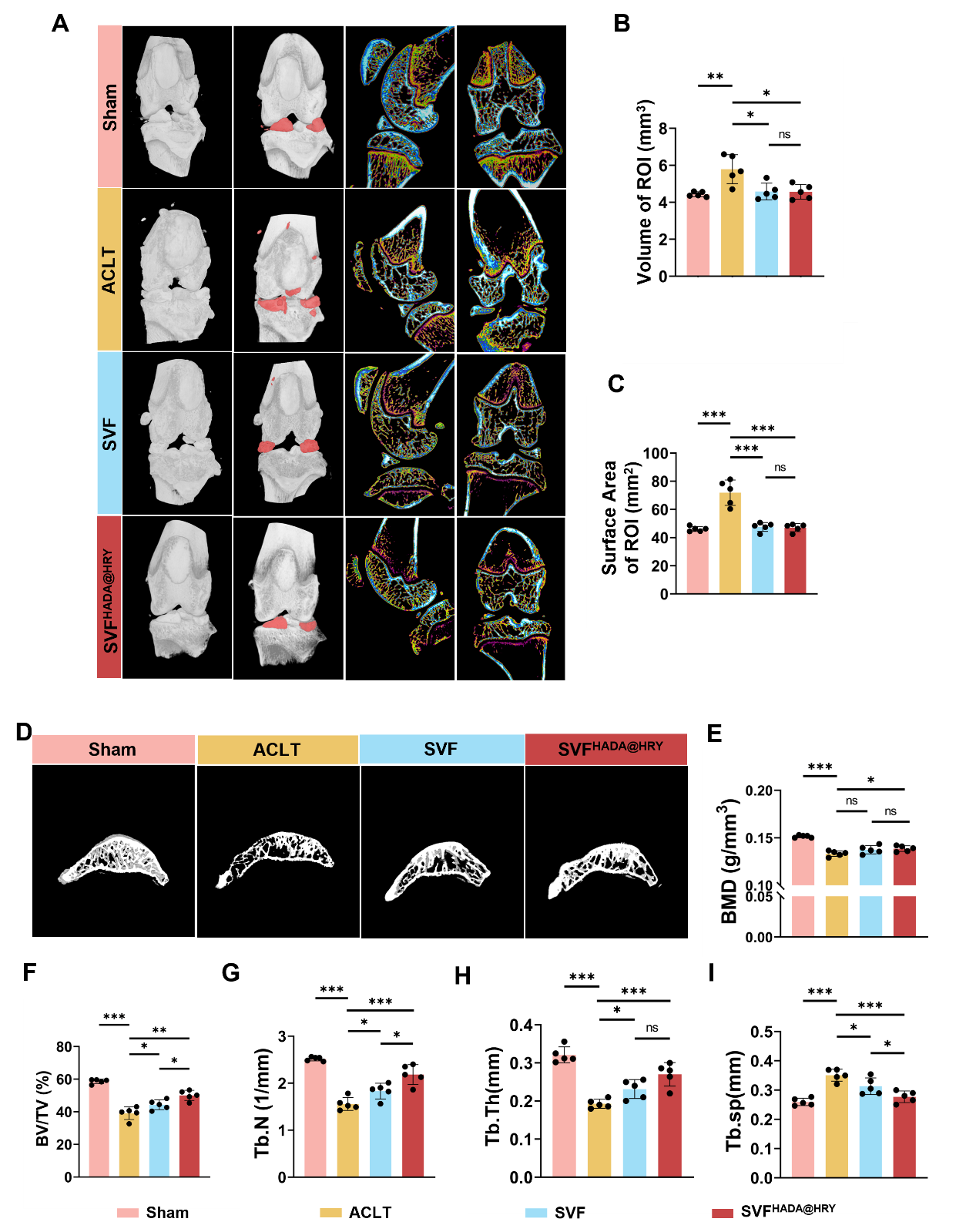
Figure S14.** Combined HADA@HRY-SVF therapy showed therapeutic effects on OA at postoperative 30 days in the ACLT model. A) Representative three dimensional (3D) micro-CT scans of knee joints at 30 days after ACLT surgery. B-C) Micro-CT based quantitative assessment of the surface area of osteophytes and the volume of the region of interest (ROI). D) Representative 3D micro-CT images of sagittal visualizations of the medial compartment's subchondral bone at 30 days following ACLT surgery. E) Quantitative micro-CT investigation of the ratio of bone mineral density (BMD) in tibial subchondral bone and trabecular bone complex. F) Bone volume to total volume (BV/TV). G) Trabecular number (Tb. N). H) Trabecular thickness (Tb. Th). I) Trabecular separation (Tb. Sp). Sham: sham-surgery. ACLT: ACLT-surgery. SVF: ACLT-surgery treated with SVF. SVF^HADA@HRY^: ACLT-surgery treated with SVF in conjunction with HADA@HRY hydrogel. n = 5 per group. All statistical data are represented as mean ± standard deviation. Statistical analyses were performed using One-way ANOVA analysis of variance with Bonferroni’s post-hoc test. **P* < 0.05, ***P* < 0.01, and ****P* < 0.001.


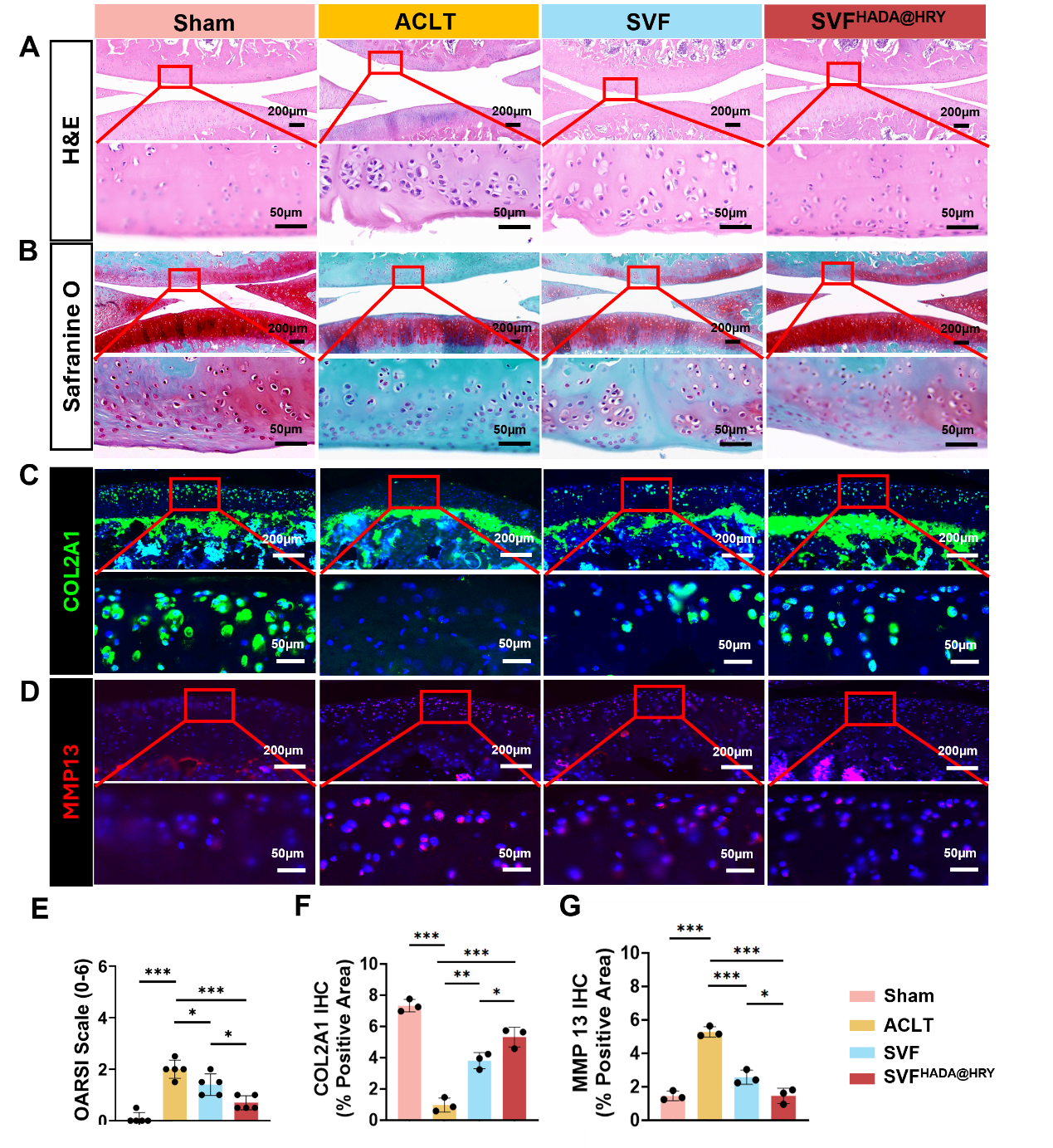


**Figure S15.** Cartilage regeneration by combining implantation of HADA@HRY hydrogel and SVF. A) Representative images of H&E-stained sagittal sections (n = 5). B) Representative images of Safranin O- and Fast Green-stained sagittal sections (n = 5). C) Immunofluorescence staining assay of Collagen Type II (Col 2A1) in knee cartilage (n = 3). D) Immunofluorescence staining assay of matrix metalloproteinase-13 (MMP-13) in knee cartilage (n = 3). ACLT: ACLT-surgery. SVF: ACLT-surgery treated with SVF. SVF^HADA@HRY^: ACLT-surgery treated with SVF in conjunction with HADA@HRY hydrogel. n = 5 per group.

**Supplementary Tables**

Table S1. Oligonucleotide primer sequences employed in reverse transcription-quantitative PCR (RT-qPCR) assays.

| Gene | Sequence (forward *5’-3’*) | Sequence (reverse *5’-3’*) |
| --- | --- | --- |
| *Oct4* | GTTGGAGAAGGTGGAACCAA | CCAAGGTGATCCTCTTCTGC |
| *Nanog* | TCGCCCTTCCTCTGAAGAC | TGCTTCTGAAACCTGTCCTTGA |
| *Klf4* | GGGAGAAGACACTGCGTCA | GGAAGCACTGGGGGAAGT |
| *Sox9* | GGCAAGCTCTGGAGACTTCTG | CCCGTTCTTCACCGACTTCC |
| *Runx2* | ACCAAGTAGCCAGGTTCAAC | GAGGATTTGTGAAGACTGTTATGG |
| *PPARg* | CCCCTACAGAGTATTACG | TCTCTCCGTAATGGAAGACC |
| *Gapdh* | CATCTCAGTCGTTCCCAAAGT | TTCCCAGGACTGGACTGT |

Table S2. Sequences of Foxm1 shRNA.

| shRNA | Sequence (forward *5’-3’*) | Antisense (reverse *5’-3’*) |
| --- | --- | --- |
| *Foxm1* sequence 1 | TCCAGCTGGAATCAAGATTAT | ATAATCTTGATTCCAGCTGGATTTTT |
| *Foxm1* sequence 2 | GGACAACAGCTTAACCAATAT | ATATTGGTTAAGCTGTTGTCCTTTTT |
| *Foxm1* sequence 3 | GCCTCTGGCAGCATCTCTTAT | ATAAGAGATGCTGCCAGAGGCTTTTT |

Table S3. Antibodies used in this study.

| Antibodies | Source | Identifier |
| --- | --- | --- |
| Mouse Anti- Foxm1 | Santa Cruz Biotechnology | Cat# Sc-376471 |
| Rabbit Anti-COL2A | Abcam | Cat# Ab-34712 |
| Rabbit Anti-MMP13 | Abcam | Cat# Ab-39012 |
| Mouse Anti-Cdkn1A | Santa Cruz Biotechnology | Cat# Sc-6264 |
| Mouse Anti-Cdkn2A | Santa Cruz Biotechnology | Cat# Sc-1661 |
| Rabbit Anti-CD73 | Cell Signaling Technology | Cat# 13160T |
| Mouse Anti-CD44 | Santa Cruz Biotechnology | Cat# Sc-7297 |
| Mouse Anti-CD29 | Santa Cruz Biotechnology | Cat# Sc-374429 |
| Mouse Anti- p-Histone H2A.X | Santa Cruz Biotechnology | Cat# sc-517348 |
| Mouse Anti -GAPDH | Proteintech | Cat# Ma-6007 |
| Anti-FITC-COL2A | Servicebio | Cat# Gb-22303 |
| Anti-CY3-MMP13 | Servicebio | Cat# Gb-21302 |

**References**

[1] R. Seino, K. Nishikubo, H. Fukunaga, Cell cycle- and dose-dependent effects on mitochondrial DNA copy number variation following irradiation, J Cell Sci (2025) jcs.263642. https://doi.org/10.1242/jcs.263642.

[2] J.D. Buenrostro, P.G. Giresi, L.C. Zaba, H.Y. Chang, W.J. Greenleaf, Transposition of native chromatin for fast and sensitive epigenomic profiling of open chromatin, DNA-binding proteins and nucleosome position, Nat Methods 10 (2013) 1213–1218. https://doi.org/10.1038/nmeth.2688.

[3] M.R. Corces, A.E. Trevino, E.G. Hamilton, P.G. Greenside, N.A. Sinnott-Armstrong, S. Vesuna, A.T. Satpathy, A.J. Rubin, K.S. Montine, B. Wu, A. Kathiria, S.W. Cho, M.R. Mumbach, A.C. Carter, M. Kasowski, L.A. Orloff, V.I. Risca, A. Kundaje, P.A. Khavari, T.J. Montine, W.J. Greenleaf, H.Y. Chang, An improved ATAC-seq protocol reduces background and enables interrogation of frozen tissues, Nat Methods 14 (2017) 959–962. https://doi.org/10.1038/nmeth.4396.

[4] X. Li, L. Jia, X. Chen, Y. Dong, X. Ren, Y. Dong, Y. Chen, L. Xie, M. Liu, C. Shiota, G.K. Gittes, L. Rui, Z. Chen, Islet α-cell Inflammation Induced By NF-κB inducing kinase (NIK) Leads to Hypoglycemia, Pancreatitis, Growth Retardation, and Postnatal Death in Mice, Theranostics 8 (2018) 5960–5971. https://doi.org/10.7150/thno.28960.
